# Advanced MR Techniques for Preoperative Glioma Characterization: Part 1

**DOI:** 10.1002/jmri.28662

**Published:** 2023-03-03

**Authors:** Lydiane Hirschler, Nico Sollmann, Bárbara Schmitz‐Abecassis, Joana Pinto, Fatemehsadat Arzanforoosh, Frederik Barkhof, Thomas Booth, Marta Calvo‐Imirizaldu, Guilherme Cassia, Marek Chmelik, Patricia Clement, Ece Ercan, Maria A. Fernández‐Seara, Julia Furtner, Elies Fuster‐Garcia, Matthew Grech‐Sollars, Nazmiye Tugay Guven, Gokce Hale Hatay, Golestan Karami, Vera C. Keil, Mina Kim, Johan A. F. Koekkoek, Simran Kukran, Laura Mancini, Ruben Emanuel Nechifor, Alpay Özcan, Esin Ozturk‐Isik, Senol Piskin, Kathleen Schmainda, Siri F. Svensson, Chih‐Hsien Tseng, Saritha Unnikrishnan, Frans Vos, Esther Warnert, Moss Y. Zhao, Radim Jancalek, Teresa Nunes, Kyrre E. Emblem, Marion Smits, Jan Petr, Gilbert Hangel

**Affiliations:** ^1^ C.J. Gorter MRI Center, Department of Radiology Leiden University Medical Center Leiden The Netherlands; ^2^ Department of Diagnostic and Interventional Radiology University Hospital Ulm Ulm Germany; ^3^ Department of Diagnostic and Interventional Neuroradiology, School of Medicine, Klinikum rechts der Isar Technical University of Munich Munich Germany; ^4^ TUM‐Neuroimaging Center, Klinikum rechts der Isar Technical University of Munich Munich Germany; ^5^ Department of Radiology Leiden University Medical Center Leiden The Netherlands; ^6^ Medical Delta Foundation Delft The Netherlands; ^7^ Institute of Biomedical Engineering, Department of Engineering Science University of Oxford Oxford UK; ^8^ Department of Radiology & Nuclear Medicine Erasmus MC Rotterdam The Netherlands; ^9^ Department of Radiology & Nuclear Medicine Amsterdam UMC, Vrije Universiteit Amsterdam The Netherlands; ^10^ Queen Square Institute of Neurology and Centre for Medical Image Computing University College London London UK; ^11^ School of Biomedical Engineering and Imaging Sciences King's College London London UK; ^12^ Department of Neuroradiology King's College Hospital NHS Foundation Trust London UK; ^13^ Department of Radiology Clínica Universidad de Navarra Pamplona Spain; ^14^ Hospital Santa Luzia Rede D'Or São Luiz Brasília Brazil; ^15^ Department of Technical Disciplines in Medicine, Faculty of Health Care University of Prešov Prešov Slovakia; ^16^ Department of Diagnostic Sciences Ghent University Ghent Belgium; ^17^ Department of Medical Imaging Ghent University Hospital Ghent Belgium; ^18^ IdiSNA, Instituto de Investigación Sanitaria de Navarra Pamplona Spain; ^19^ Department of Biomedical Imaging and Image‐guided Therapy Medical University of Vienna Vienna Austria; ^20^ Research Center of Medical Image Analysis and Artificial Intelligence Danube Private University Krems an der Donau Austria; ^21^ Biomedical Data Science Laboratory, Instituto Universitario de Tecnologías de la Información y Comunicaciones Universitat Politècnica de València Valencia Spain; ^22^ Centre for Medical Image Computing, Department of Computer Science University College London London UK; ^23^ Lysholm Department of Neuroradiology, National Hospital for Neurology and Neurosurgery University College London Hospitals NHS Foundation Trust London UK; ^24^ Institute of Biomedical Engineering Bogazici University Istanbul Istanbul Turkey; ^25^ Cancer Center Amsterdam Amsterdam The Netherlands; ^26^ Centre for Medical Image Computing, Department of Medical Physics & Biomedical Engineering and Department of Neuroinflammation University College London London UK; ^27^ Department of Neurology Leiden University Medical Center Leiden The Netherlands; ^28^ Department of Neurology Haaglanden Medical Center The Hague The Netherlands; ^29^ Department of Bioengineering Imperial College London London UK; ^30^ Department of Radiotherapy and Imaging Institute of Cancer Research London UK; ^31^ Department of Brain Repair and Rehabilitation, Institute of Neurology University College London London UK; ^32^ Department of Clinical Psychology and Psychotherapy International Institute for the Advanced Studies of Psychotherapy and Applied Mental Health, Babes‐Bolyai University Cluj‐Napoca Romania; ^33^ Electrical and Electronics Engineering Department Bogazici University Istanbul Istanbul Turkey; ^34^ Department of Mechanical Engineering, Faculty of Natural Sciences and Engineering Istinye University Istanbul Istanbul Turkey; ^35^ Department of Biophysics Medical College of Wisconsin Milwaukee Wisconsin USA; ^36^ Department of Physics and Computational Radiology Oslo University Hospital Oslo Norway; ^37^ Department of Physics University of Oslo Oslo Norway; ^38^ Department of Imaging Physics Delft University of Technology Delft The Netherlands; ^39^ Faculty of Engineering and Design Atlantic Technological University (ATU) Sligo Sligo Ireland; ^40^ Mathematical Modelling and Intelligent Systems for Health and Environment (MISHE), ATU Sligo Sligo Ireland; ^41^ Department of Radiology Stanford University Stanford California USA; ^42^ Stanford Cardiovascular Institute Stanford University Stanford California USA; ^43^ Department of Neurosurgery St. Anne's University Hospital, Brno Brno Czech Republic; ^44^ Faculty of Medicine, Masaryk University Brno Czech Republic; ^45^ Department of Neuroradiology Hospital Garcia de Orta Almada Portugal; ^46^ Brain Tumour Centre Erasmus MC Cancer Institute Rotterdam The Netherlands; ^47^ Helmholtz‐Zentrum Dresden‐Rossendorf Institute of Radiopharmaceutical Cancer Research Dresden Germany; ^48^ Department of Neurosurgery Medical University of Vienna Vienna Austria; ^49^ High Field MR Centre, Department of Biomedical Imaging and Image‐guided Therapy Medical University of Vienna Vienna Austria; ^50^ Christian Doppler Laboratory for MR Imaging Biomarkers Vienna Austria; ^51^ Medical Imaging Cluster Medical University of Vienna Vienna Austria

**Keywords:** glioma, brain, preoperative, contrasts, GliMR 2.0, level of clinical validation

## Abstract

Preoperative clinical magnetic resonance imaging (MRI) protocols for gliomas, brain tumors with dismal outcomes due to their infiltrative properties, still rely on conventional structural MRI, which does not deliver information on tumor genotype and is limited in the delineation of diffuse gliomas. The GliMR COST action wants to raise awareness about the state of the art of advanced MRI techniques in gliomas and their possible clinical translation or lack thereof. This review describes current methods, limits, and applications of advanced MRI for the preoperative assessment of glioma, summarizing the level of clinical validation of different techniques. In this first part, we discuss dynamic susceptibility contrast and dynamic contrast‐enhanced MRI, arterial spin labeling, diffusion‐weighted MRI, vessel imaging, and magnetic resonance fingerprinting. The second part of this review addresses magnetic resonance spectroscopy, chemical exchange saturation transfer, susceptibility‐weighted imaging, MRI‐PET, MR elastography, and MR‐based radiomics applications.

Evidence Level: 3

Technical Efficacy: Stage 2

Gliomas are a heterogeneous group of neuroepithelial tumors arising from the glial cells, with an age‐adjusted average rate of 6.03 per 100,000 population.^1^ Traditionally, they are divided according to a four‐step grading system where a higher grade represents disease with more malignant features and a mostly dismal prognosis. The traditional concept of the World Health Organization (WHO) grading system based on histopathological assessment underwent significant changes in the fifth edition of the WHO Classification of Tumors of the Central Nervous System (CNS), published in 2021.^2^ This current classification introduced revisions to tumor nomenclature and advances the integral role of molecular diagnostics for tumor classification and grading that predicts the prognosis better^3^ than the previous 2016 version.^4^

Compared with the 2016 version, the WHO 2021 classification incorporates more molecular alterations into the diagnostics and divides gliomas into adult‐type diffuse gliomas, pediatric‐type diffuse low‐grade (LGG) and high‐grade (HGG) gliomas, circumscribed astrocytic gliomas, glioneuronal and neuronal tumors, and ependymal tumors. The primary genetic markers used for glioma taxonomy are now considered isocitrate dehydrogenase (IDH) 1 and 2 mutation status, 1p/19q co‐deletion, H3F3A alterations, ATRX gene mutations, O6‐Methylguanine‐DNA Methyltransferase (MGMT) promoter methylation status, loss of CDKN2A, epidermal growth factor receptor (EGFR) amplification, a combined gain of chromosome 7 and loss of chromosome 10, and TERT promoter pathogenic variants. In adults, the term glioblastoma is now reserved only for IDH‐wildtype tumors and will always be graded as 4, whereas IDH‐mutated astrocytomas present a distinct progressive disease with WHO grade rising from 2 to 4. As a third class, oligodendrogliomas are now distinct from astrocytomas by possessing both IDH mutation and 1p/19q co‐deletion and can range form grade 2 to 4 as well. As the genetic profile of a particular tumor affects its metabolic pathways leading to a certain product or a change in the cell's phenotype, advanced magnetic resonance imaging (MRI) techniques can be a very promising noninvasive approach to predict glioma type and behavior.

Preoperative glioma imaging by MRI is essential to localize and delineate the tumor volume and to assess infiltrative behavior or compressive effects on adjacent structures with related complications. The minimal recommendation for such routine structural imaging protocols at 3T consists of T1‐weighted imaging (before and after the administration of gadolinium‐based contrast agents (GBCA), 1 mm isotropic resolution), T2‐weighted imaging (after GBCA administration, <3 mm slice thickness), T2‐weighted fluid‐attenuated inversion recovery imaging (<3 mm slice thickness), and diffusion‐weighted imaging (<3 mm slice thickness, *b*‐values of 0, 500, and 1000 s/mm^2^), with further details to be found in Ellingson et al.^5^

With the advent of advanced sequences, quantitative imaging of multiple pathophysiological features in the tumor and surrounding tissue became possible,^6^, ^7^ providing the opportunity to noninvasively characterize different molecular types of glioma against the background of the WHO 2021 classification.^6^, ^8^ While glioma genotyping based on tissue probes derived from neurosurgical tumor resection or biopsy remains the standard, predicting genotypes by preoperative advanced MRI could aid in clinical decision‐making and facilitate individual management tailored to the individual tumor characteristics.^6^, ^9^

In most clinical settings for preoperative glioma assessment, however, only conventional MRI is performed. The untapped potential of advanced MRI seems related to a multitude of obstacles that prevent its wider translation into the clinical routine.^10^ A major hurdle is the lack of rigorous validation of advanced MRI‐derived biomarkers. Although recommendations for the acceleration of imaging biomarker development in cancer, both for lesion segmentation and imaging biomarker quantification, do exist, almost no regulatory qualifications or specific guidelines of high quality have been adopted.^11^, ^12^, ^13^ Finally, advanced sequences beyond conventional structural MRI may require special hardware and/or software combined with the need for dedicated expertise for acquisition, post‐processing, and evaluation.^14^ This makes advanced imaging of gliomas time‐consuming, often involving manual data handling and dedicated, custom‐made processing pipelines.

The purpose of this review is to raise awareness and contribute to clinical translations of advanced MRI techniques by describing the methods and application of different modalities for the preoperative assessment of glioma, and summarizing whether these techniques can be routinely used. The first part of this review includes perfusion imaging by dynamic contrast‐enhanced (DCE), dynamic susceptibility contrast (DSC), and arterial spin labeling (ASL), as well as diffusion MRI, vessel imaging, and relaxometry and MR fingerprinting (MRF). The second part of this review describes MR spectroscopy, chemical exchange saturation transfer (CEST), susceptibility‐weighted imaging (SWI), MRI‐PET, MR elastography (MRE), and MR‐based radiomics and artificial intelligence (AI) applications. For each technique, we aimed to provide a concise methodological overview, review the strengths and weaknesses of glioma characterization and tumor heterogeneity mapping, and use this as the basis for assessing the level of technical readiness of each method.

## Methods

This review was initiated through the European Cooperation in Science and Technology (COST) Glioma MR Imaging 2.0 (GliMR) initiative,^10^ which brought together clinicians, engineers, and physicists with expertise in advanced MRI techniques applied to brain tumor imaging in a series of virtual and onsite meetings from July 2020 through September 2022. We defined the target audience of this review as clinicians (eg, neuroradiologists, neurosurgeons, and (neuro‐)oncologists) and researchers without deep knowledge of advanced MRI who want to broaden their routine or experimental protocols for brain tumor imaging. We used the GliMR consortium's technical expertise to aggregate the available evidence and level of validation for cutting‐edge MRI methods and the information derivable from these.

These advanced MRI techniques allow (semi)quantitative imaging of tumor composition, metabolism, physiology, or mechanical properties that are not captured in routine clinical protocols. At the same time, we included only acquisition, reconstruction, and postprocessing methods that have already demonstrated pilot results in brain tumors.

As this review cannot be a complete review of the literature, all topical experts were instructed to select recent peer‐reviewed publications indexed in the MEDLINE database, including reviews that described the techniques with the highest potential impact. Together with a brief technological introduction for every topic, this was intended as a base for our evaluation of the methodological readiness of preoperative advanced MRI methods for future clinical practice.

The methodologies/contrasts within the scope of the work were determined as DCE/DSC, ASL, diffusion MRI, vessel imaging, and MRF—included in the first part of the review—and MRS, CEST, SWI, MR‐PET, MRE, and MR‐based radiomics—included in the second part. The reviews for these specific sequences were designed to include the following:

- An *Overview* of the technique with links to detailed reviews and recommendations for implementation and use;
- An overview of the current evidence about the *Clinical application* to brain tumor imaging, focusing on how it can be used for glioma characterization and grading according to the new WHO 2021 classification criteria and its focus on molecular characteristics to distinguish between different molecular glioma subtypes, namely oligodendroglioma, IDH‐mutant, and 1p/19q co‐deleted, astrocytoma, IDH‐mutant, and glioblastoma, IDH‐wildtype, as well as the improved mapping of structural, functional, and metabolic glioma heterogeneity;
- A statement on the level of clinical and technological *Validation* of the method, summarizing the current status and the prospect for near‐future improvements;
- A *Summary* of the recommended use.

The expert panel composed of the authors has addressed the level of validation of all techniques. First, a survey was sent to all the experts, which included questions on the acquisition, processing, and clinical evidence of each method for pre‐treatment glioma characterization. If there was a lack of consensus in the answers, the expert panel reviewed the recent literature. After multiple consensus meetings, the level of validation of each technique was summarized in a table (Table 1) that included relevant literature references. Table 2 summarizes specifically the clinical applications of all presented MRI methods for the prediction of molecular glioma subtypes defined in the WHO 2021 classification.

**TABLE 1:** Level of validation table

	Track & Domain^a^	Perfusion			Diffusion		VSI	MRF	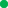	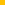	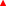	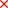
		DSC	DCE	ASL	ADC	DTI						
Technical validation												
Test–retest repeatability	T2	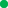	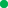	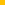	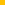	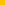	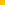	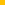	Yes, with current standard implementation	Yes, but with other implementation or patient group/animal model	None available	Unclear
Cross‐vendor reproducibility	T2	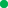	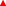	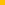	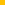	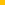	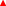	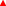	Yes, with current standard implementation	Yes, but with other implementation or patient group	None available	Unclear
Multi‐site reproducibility	T3	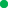	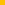	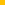	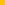	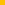	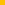	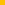	Yes, with current standard implementation	Yes, but with other implementation or patient group, phantom or analysis	None available	Unclear
Clinical evidence												
Proof‐of‐concept in patients	C1	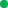	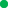	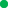	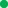	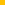	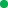	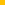	Differentiation tumor types/grades	Differentiation of tumor from normal brain	None available	Unclear
Evaluated in clinical studies	C2‐3	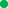	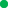	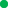	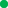	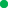	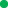	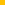	Multiple single center	Few or preliminary studies	None available	Unclear
Evaluated in multi‐center studies	C3	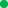	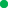	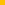	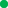	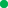	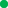	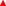	Good quality with relevant question	Small, preliminary or only method stability/not relevant question	None available	Unclear
Evaluated in meta‐analysis		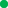	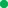	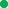	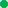	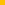	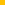	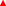	Consistent result with standard measure	Not standard measure/method, or low number of studies/patients	None available	Unclear
Established diagnostic accuracy, cut‐offs/criteria	C3	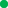	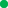	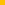	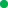	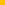	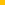	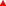	Consistent in multiple single‐center studies	Few or preliminary studies	None available	Unclear
Acceptance												
Method guidelines recommendations	T	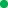	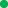	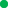	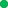	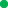	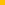	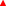	Available and updated	Available, but not updated or not specific for tumor imaging	None available	Unclear
Included in national imaging guidelines		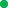	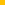	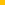	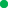	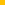	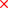	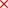	Endorsed by a majority of the community	Only endorsed by a minority	Not mentioned	Unclear
Included in clinical trial guidelines^b^		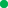	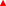	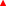	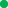	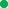	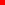	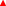	Included in suggested standard protocol	Mentioned, but clinical value uncertain	Not mentioned	Unclear
Included in international clinical guidelines^c^		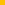	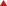	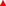	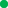	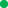	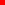	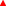	Endorsed by major international society guidelines	Mentioned, but clinical value uncertain	Not mentioned	Unclear
In clinical use for brain tumor imaging^d^		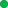	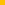	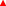	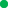	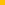	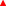	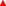	Widely implemented (>50%)	Intermediate (<50%)	Uncommon	Unclear
In clinical use for glioma diagnosis^d^		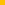	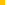	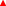	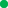	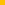	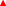	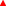	Widely applied (>50%)	Intermediate (<50%)	Uncommon	Unclear
Implementation												
Sequence availability	T2	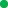	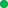	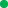	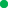	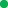	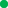	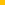	Comparable sequence available as clinical from all major vendors	No standard implementation or only WIP	Research sequence at singles sites	Unclear
Post‐processing software availability	T2	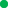	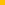	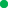	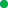	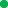	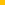	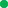	On‐line scanner/reading workstation with best practice implementation	Off‐line, commercially available software	In‐house software	Unclear
Subjective ease of data acquisition (scanner operator)	T2	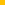	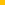	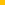	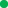	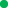	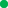	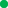	Minimal need for training	Special training/attention required	Difficult to obtain good quality data	Unclear
Subjective ease of post‐processing (within clinical department)		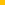	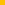	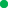	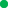	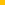	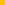	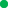	No post‐processing needed	Extra processing/training needed, but not time consuming	Expert or time intensive processing required	Unclear
Subjective ease of data interpretation (clinician)		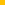	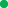	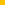	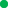	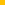	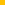	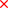	Visual reading or only simple manual steps required	Special training/expertise required	Highly specialized in single centers	Unclear

(a) Imaging biomarker roadmap; (b) RANO, iRANO, Standardized Brain Tumor Imaging Protocol; (c) GBM EANO/SNO, EANO diff. glioma, EANO glioma; (d) European survey of advanced MRI, US survey of perfusion imaging. T technical validation C clinical validation, Domain 1 Discovery, Domain 2 Validation, Domain 3 Qualification. References to the guidelines and also further material for each technique are included in the supplementary materials.

**TABLE 2:** Summary of clinical applications for the prediction of molecular subtypes in gliomas as presented in this review

Methodology	Parameters	Molecular Marker	References
DSC	rCBV	IDH mutation	Lu (2021)
DSC	rCBV	MGMT methylation	Lu (2021)
DCE	*K* ^trans^, *V*_e_	IDH mutation	Hu (2020)
DCE	*K* ^trans^	MGMT methylation	Zhang (2017)
ASL	CBF	IDH mutation	Yoo (2020)
ASL	CBF	MGMT methylation	Yoo (2020)
ASL	CBF	p53 expression	Mao (2020)
DWI	ADC	IDH mutation	Leu (2017); Wang (2021)
DWI	ADC	1p/19q co‐deletion	Leu (2017)
VAI	CMRO2, MTI	IDH mutation	Stadlbauer (2017)
MRF	T2	IDH mutation	Kern (2020)
MRF	T1	IDH mutation	Springer (2022)

## Results

### DSC‐MRI

#### OVERVIEW

**FIGURE 1:**
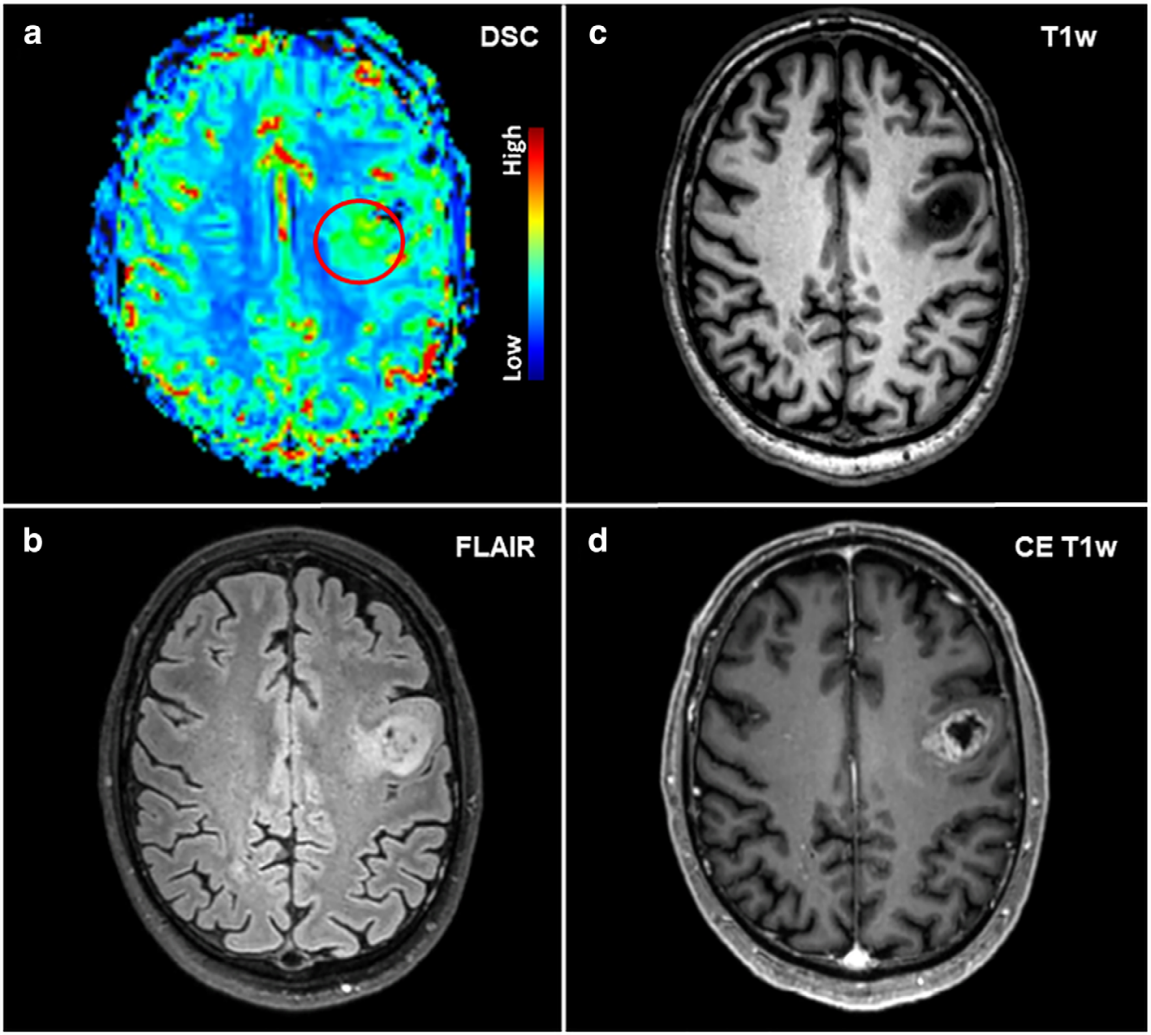
Elevated perfusion according to dynamic susceptibility contrast (DSC) MRI (**a**) in a 55‐year‐old male patient with a left frontal high‐grade glioma (HGG) that showed high signal on fluid‐attenuated inversion recovery (FLAIR); (**b**) imaging and contrast enhancement on T1‐weighted imaging (**c**, axial non‐contrast, and **d**, axial contrast‐enhanced images). The borders of the lesion with contrast‐enhancing tumor parts, in particular, showed hyperperfusion on DSC MRI (red circle, a).

DSC‐MRI entails the acquisition of T2 or T2*‐weighted images with a high temporal resolution during which a GBCA is bolus‐injected. A gradient‐echo echo planar imaging (GRE‐EPI) sequence, heavily T2*‐weighted, is most often used. With GBCA confined to the vessels, as for the brain with an intact blood‐brain barrier (BBB), a gradient of susceptibility between the intra‐ and extravascular tissue is induced, causing a transient shortening of the dynamic T2*‐weighted signal (S(t)). The S(t) is converted into the relaxation rate change (∆R2 × (t)), which, when integrated (added up), provides a voxelwise estimate of the relative cerebral blood volume (rCBV) (relative to the rest of the brain). In addition, voxelwise cerebral blood flow (CBF) can be estimated if the ∆R2 × (t) from large arteries (i.e., the arterial input function [AIF]) is also separately measured and used, along with the tissue ∆R2 × (t). Since rCBV is the most common DSC‐MRI parameter used to evaluate brain tumors (Fig. 1), the remaining discussion will focus on rCBV.

Estimation of rCBV can be confounded by the extravasation of GBCA through a disrupted BBB, a common condition in brain tumors. While this “leakage effect” violates the assumption of GBCA vascular compartmentalization, DSC‐MRI can still be successfully used to estimate brain tumor rCBV if this leakage effect is appropriately considered.^15^, ^16^, ^17^ A recent consensus on DSC‐MRI data acquisition for brain tumors resulted in two recommended approaches.^18^ The first, and most robust approach incorporates a GBCA pre‐dose to diminish T1 leakage effects that might occur during the subsequent DSC‐MRI acquisition. A second GBCA dose is administered during the collection of the DSC‐MRI data, using either a low (30°) or intermediate (60°) flip angle and field strength‐dependent TEs (40–50 msec at 1.5T, 25–35 msec at 3T). The second approach has the advantage of not requiring a GBCA pre‐dose while using a low flip angle (30°) and field‐strength‐dependent TEs (1.5T: 40–50 msec; 3T: 25–35 msec). For both approaches, a TR = 1000–1500 msec is recommended, and the inclusion of a post‐processing, the contrast‐agent leakage correction method is required. While the Boxerman‐Schmainda‐Weiskoff (BSW) method^16^ for leakage correction is most commonly used, other methods have also been proposed.^19^, ^20^, ^21^

#### CLINICAL APPLICATION

Studies have shown that rCBV ratios can predict glioma grade^15^, ^22^, ^23^, ^24^ and are able to stratify patients into low, intermediate, and high‐risk groups, with shorter survival corresponding to higher rCBV.^25^ Both intra‐tumoral and peri‐tumoral rCBV were shown to be reliable for the preoperative distinction of HGG from LGG with excellent sensitivity and accuracy.^26^ Similarly, delineations of pre‐operative rCBV “habitats” within both contrast‐enhancing and peritumoral regions were found to be highly prognostic for patients who underwent standard‐of‐care treatment.^27^

**FIGURE 2:**
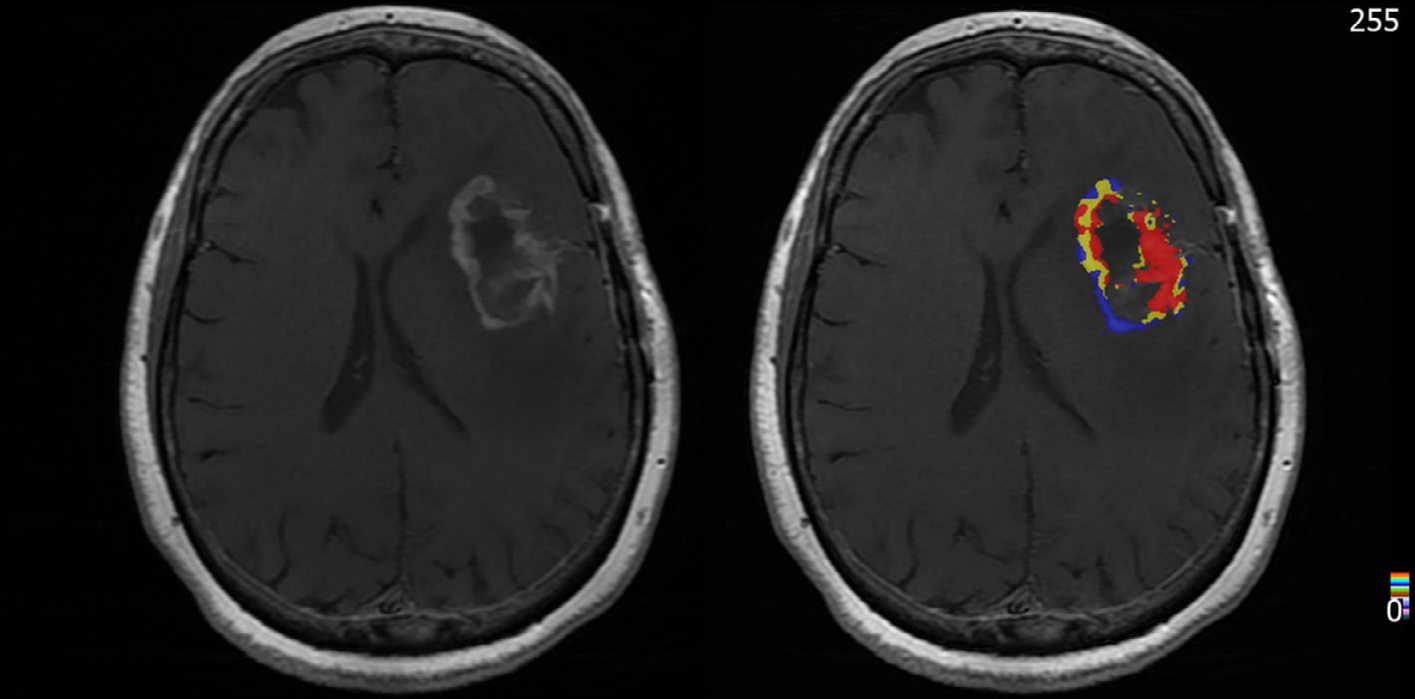
Patient with recurrent glioblastoma. (**a**) T1w MRI with CEA, (**b**) corresponding map of fractional tumor burden (FTB) showing regions of zero‐low (blue), intermediate (yellow), and high rCBV (red)within the contrast agent enhancing region.

Possibly, one of the most significant roles of pre‐operative rCBV is to assist with ensuring an accurate diagnosis as the heterogeneity of gliomas can lead to misdiagnosis and undergrading. Brain tumor rCBV has been shown helpful in identifying such cases retrospectively,^25^ or, preferably, both can be avoided altogether by identifying the best sites for surgical biopsy.^28^ In a more recent case report,^29^ rCBV class maps (referred to as fractional tumor burden maps), which delineate regions of low, intermediate, and high vascularity (Fig. 2), confirmed that tissue obtained from areas of zero to low rCBV received a histopathologic diagnosis of non‐tumor while the remaining unresectable tissue, with a high pre‐operative rCBV, was the site of early and aggressive recurrence. Thus, knowledge of the spatial variation in rCBV in both resected and the remaining tissue is fundamental for an accurate diagnosis and follow‐up treatment management.

Pre‐operative rCBV may also play an important role in the success of the 2016 WHO classification that newly includes molecular markers. Despite the known heterogeneities, at both the cellular and molecular levels, patient stratification and treatment are generally determined on the basis of molecular markers present in a single tumor specimen. As a result, the power of this new classification is being profoundly underutilized and may explain why, even with the advances of molecular profiling, the improvements in patient outcome have been modest.^30^ As a potential solution, rCBV was able to predict differences in IDH1 mutation and MGMT status,^31^ and tissue from hypercellular and hypervascular microfoci revealed greater expression of Ki‐67, HIF‐1a, CD31, and EGFR compared to tumor background.^32^ Therefore, rCBV has the potential to guide surgical biopsy and provide a more accurate diagnosis for both histopathological and molecular analyses.

#### VALIDATION

Existing evidence reveals that rCBV is a valuable, and even necessary adjunct to standard MRI. Yet, it has been argued that rCBV remains limited in its clinical adoption due to a lack of standardization, which may explain the variability in reported rCBV thresholds.^33^ Still, in recent years several well‐curated studies have demonstrated excellent repeatability, cross‐site consistency, and market availability, suggesting a high technology readiness level for rCBV.

With DSC‐MRI data collected twice within 8 days, in HGG patients, rCBV was found to be highly repeatable.^34^ The within‐patient coefficient of variation was further reduced when using a standardization algorithm that precluded the need for a user‐defined reference region, which is required to normalize rCBV to normal brain values. Similar results were found in a multi‐site clinical trial, for which rCBV repeatability was again shown to be excellent with standardized rCBV more repeatable than normalized rCBV.^35^

With multi‐site analysis of a shared DSC‐MRI dataset, but using several different analysis platforms, rCBV was also able to distinguish high‐grade from low‐grade glioma in all cases.^36^ Moreover, a single threshold, applicable to all platforms, could be identified. This study further suggested that much of the previous variability in reported thresholds may be due to differences in data pre‐processing, patient populations, or image acquisition settings, variables that were held fixed in this multi‐site study. Moreover, widespread implementation of the recommended acquisition protocol^18^ could greatly improve consistency in reported rCBV data, including thresholds by which to distinguish tumor grades. Indeed, two independent sites, using the same acquisition and post‐processing methods, were able to arrive at the same threshold to distinguish tumor from treatment effect, validated with spatially matched biopsies.^37^, ^38^

Finally, FDA‐cleared and CE‐marked platforms for the analysis of rCBV data are now widely available, with studies published that compared platforms.^36^, ^39^, ^40^ Using one such platform, the ease‐of‐use and ability to collect and analyze multi‐site rCBV data were demonstrated by incorporation into clinical trials, with each showing the utility of rCBV to predict outcomes.^35^, ^41^, ^42^

Challenges that remain for DSC‐MRI include optimization of the imaging method itself. For example, GRE‐EPI can experience signal dropout in regions near air‐tissue interfaces, bone, or resection cavities, making it difficult to evaluate tumors in these regions fully. Technical improvements that enable higher spatial resolution imaging and reduced sensitivity, or correction of the unwanted susceptibility effects, are needed. Also, GRE‐EPI retains a high sensitivity to large normal vessels, which can make it difficult to evaluate tumor‐specific vascularity in these regions. Approaches that combine GRE‐EPI and SE‐EPI^15^, ^17^, ^24^, ^43^ may be a solution, as this could offer images with differing vessel size sensitivity and a more complete interpretation. However, such sequences are not yet available for clinical use. Finally, the high temporal resolution required for DSC‐MRI often precludes whole‐brain imaging. Newer methods that incorporate advances in parallel and simultaneous multi‐slice imaging may offer a solution.^44^

#### SUMMARY

The collection of pre‐operative DSC‐MRI data with the generation of rCBV maps is easy to obtain and has been shown to be invaluable for the diagnosis and treatment management of glioma. Full clinical adoption should be accelerated with the recent consensus recommendation for DSC‐MRI data acquisition and convergence of analysis methods, thus overcoming previous concerns regarding standardization. The remaining issues include improving image quality and coverage.

### DCE

#### OVERVIEW

**FIGURE 3:**
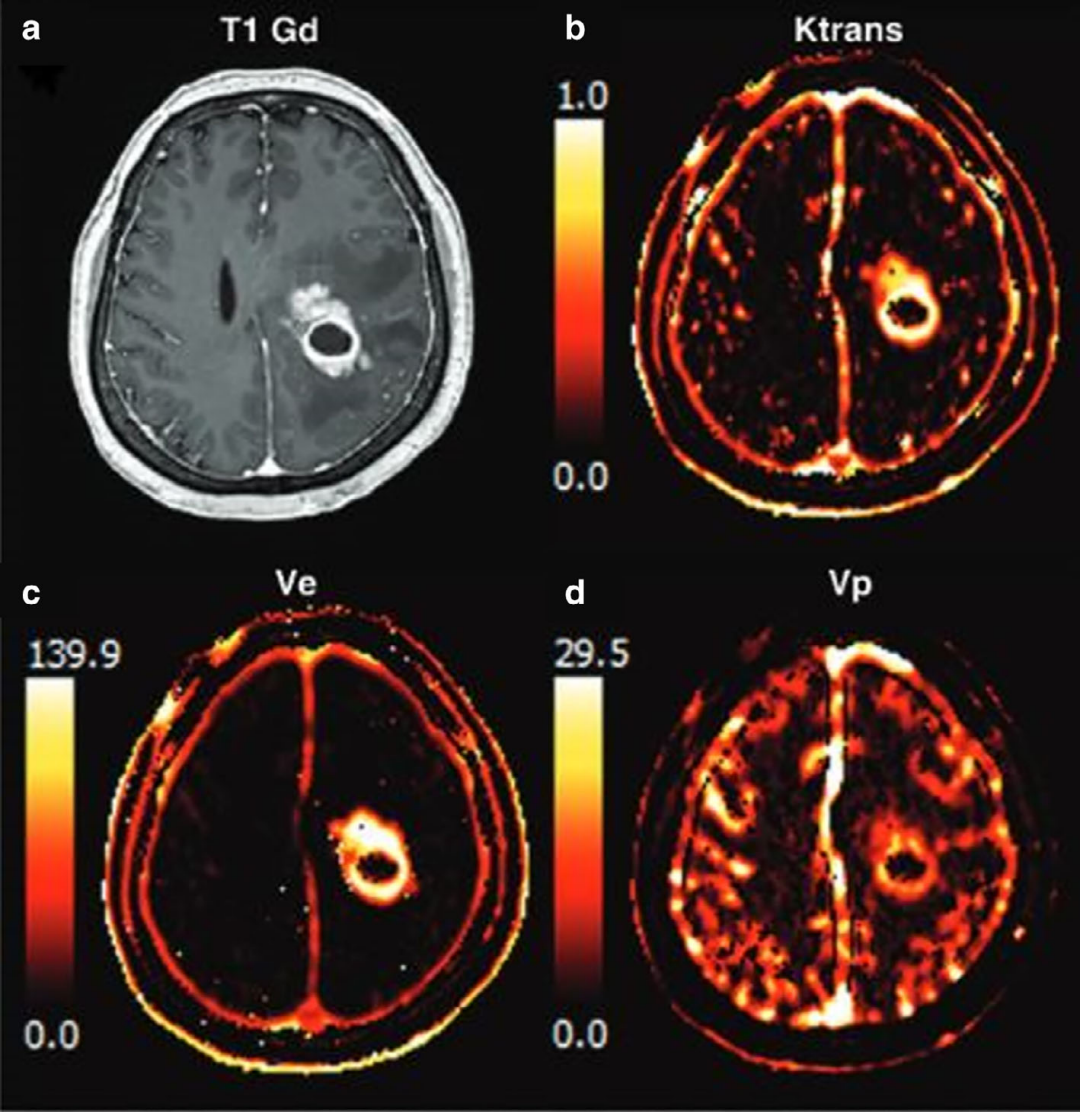
The contrast‐enhanced T1‐weighted image (**a**) and dynamic contrast‐enhanced‐derived vascular parameter maps: *K* ^trans^ (**b**), *V*_e_ (**c**), and *V*_p_ (**d**) of a glioblastoma patient treated with concurrent radiation therapy and temozolomide chemotherapy.^47^

Dynamic contrast‐enhanced MRI (DCE‐MRI) is a perfusion technique that monitors the GBCA‐induced T1‐shortening effect in blood plasma and tissue, if leakage occurs. The signal records mixed information about blood perfusion, vessel permeability, and a fraction of extracellular extravascular space (EES), and is often used to characterize tumor microvasculature. The signal can be assessed semi‐quantitatively by evaluating the contrast arrival time, time to the peak, maximum intensity, the area under the curve, wash‐in slope, and wash‐out rate. Alternatively, a quantitative analysis is achieved by applying tracer kinetic models.^45^ The most frequently applied model in tumor assessment is the extended Tofts model, which asserts that the contrast tracer distributes over two compartments: the intravascular space and the EES, with a bi‐directional exchange of the tracer across the blood vessel wall.^46^ The model enables numerical estimation of the volume transfer constant between the blood plasma and the EES (*K* ^trans^), the reflux exchange rate from the EES to the blood plasma (*K*_ep_), the volume fraction of plasma (*V*_p_), and the volume fraction of EES (*V*_e_) (Fig. 3). The volume transfer constant, *K* ^trans^, which reflects the vascular permeability, is the most often applied DCE parameter in the context of glioma.^48^ General guidelines for applying DCE imaging in pre‐clinical research have been summarized in multiple papers.^49^, ^50^

#### CLINICAL APPLICATION

Malignant gliomas are characterized by a remarkable increase in blood vessel formation (angiogenesis) which leads to aberrant vascular structure, abnormal blood flow, and increased permeability in vessels. DCE‐driven parameters were investigated to be potential markers of angiogenic activity in gliomas and are therefore being used for tumor monitoring.^51^ An extended systematic review^52^ summarized 14 studies about the discrimination between LGGs and HGGs and five studies about the differentiation between primary CNS lymphomas and HGGs based on DCE parameters. The paper concluded that all these studies demonstrated considerable specificity and sensitivity in relation to the studied aspects, showing high diagnostic accuracy in discriminating between LGGs and HGGs (AUC 0.96) and slightly lower performance for discriminating between primary CNS lymphomas and HGGs (AUC 0.86).

Moreover, studies revealed that DCE‐driven parameters were able to predict some of the molecular characteristics used recently for the classification of glioma tumors, including IDH and MGMT methylation. Hu et al reported statistically significant differences in histogram parameters of *K* ^trans^ and *V*_e_ between IDH‐mutated and IDH‐wild‐type glioma.^53^ Furthermore, Zhang et al found that glioblastoma with MGMT methylation showed significantly higher *K* ^trans^, indicating that MGMT methylation may be involved in glioma‐associated angiogenesis characterized by high endothelial permeability vasculatures.^48^ The prognostic value of DCE parameters has also been studied, with some studies showing higher *K* ^trans^ and *V*_e_ to be associated with worse overall survival (OS), and Ulyte et al showing that high *V*_e_ is a consistent predictor of worse progression‐free survival and OS in HGG patients.^54^

#### VALIDATION

DCE MRI has been studied for over three decades. An overwhelming amount of papers have demonstrated the importance of DCE MRI for diagnosis, prognosis, and therapy monitoring in glioma patients. However, one limitation is that the DCE parameters may vary across vendors and systems, hindering cross‐center comparison. The variability of DCE parameters results from several factors including different field strengths, imaging principles, sequence settings, and analysis software. Kim has discussed the sources of variability in quantitating DCE parameters and proposed several possible solutions.^55^ Hence, a consensus for the implementation of DCE imaging with reduced bias across multi‐centers is still needed to facilitate the integration of the DCE technique into standard‐of‐care imaging in the clinic.

The selection of pharmacokinetic models is also a key factor that influences the DCE parameters. Complex models with fewer assumptions are physiologically more reliable than simpler models, which often make assumptions to constrain the model. Such assumptions may not be appropriate and could bias estimated parameters from the model. Conversely, complex models are more sensitive to noise than simpler models.^56^

#### SUMMARY

Preclinical and clinical studies have shown that the quantitative DCE MRI parameters could be image biomarkers in glioma imaging. However, it is not yet possible to use DCE MRI as a regular tool in the clinic due to the variability resulting from differences in scanners, sequences, and software. Besides, improving the acquired DCE image quality would facilitate the implementation of complex models, which are more realistic in pathological conditions, and further provides more reliable and precise DCE parameters.

### ASL

#### OVERVIEW

ASL magnetically labels arterial blood water by an inversion pulse proximal to the imaging region. After a short delay on the order of seconds, called the post‐labeling delay, an image is acquired in the brain, which will be affected by the inflow of inverted spins in blood. The difference between an image acquired with and without this labeling subtracts the tissue signal and only contains the signal from the inflowing blood, allowing quantification of the volume of labeled blood delivered to each voxel.^57^ To gain sufficient signal, the measurement has to be repeated several times.

This technique allows absolute quantification of CBF, without the need for the administration of an intravenous contrast media.^57^ Given its noninvasive nature, ASL can be easily repeated, implemented in conventional MR protocols, and used in specific populations, such as children, patients with allergic reactions to gadolinium, and patients with kidney failure, where the injection of GBCA would be contraindicated. ASL‐MRI has shown good repeatability both within sessions and between sessions with sessions days to months apart.

#### CLINICAL APPLICATION

Multiple studies have shown the role of ASL in diagnosis, grading, and preoperative planning.^58^ In general, ASL detects increased blood flow in glioma^59^ and has a high diagnostic value in distinguishing glioma from metastases, primary CNS lymphoma, and nonneoplastic brain lesions.^60^

Two meta‐analyses indicated that ASL may be useful in distinguishing HGG and LGG,^61^, ^62^ with the maximum tumor blood flow relative to contralateral healthy tissue demonstrating the best discrimination performance.^59^ HGG typically exhibits high perfusion and vascularity, consistent with the higher metabolism of the tumor tissue, and shows an above‐average signal on ASL. In contrast, LGG tends to demonstrate lower‐than‐average blood flow (with pilocytic astrocytoma and ganglioglioma the exceptions).^58^, ^60^ In addition, ASL has been reported to predict IDH1, MGMT promoter methylation (i.e., higher perfusion), and p53 status (i.e., lower perfusion),^63^, ^64^ and appears to be correlated with tumor microvascular density^65^ and VEGF expression.^66^ Nonetheless, more studies related to the specific classification of gliomas with ASL are needed.^59^ Finally, ASL can be used for prognosis prediction of patients with glioblastoma, as low‐perfused gliomas appear to be associated with longer event‐free and OS.^64^ Moreover, malignant progression in patients with grade 2 glioma can be predicted using ASL.^67^

#### VALIDATION

The 2015 ISMRM Perfusion Study Group ASL recommendations resulted from a major effort to standardize ASL for clinical applications in the brain.^57^ The suggested 3D pseudo‐continuous sequence with background suppression has been adopted by all major scanner vendors and is still considered optimal for ASL in glioma. Labeling duration of 1800 msec, post‐labeling delay 2000 msec, basic subtraction calculation, and normalization to contralateral GM values are recommended (Lindner et al MRM 2022, in revision, reference will be added during this manuscript's revision). More research is needed to confirm the added value of multiple post‐labeling delays.^68^

**FIGURE 4:**
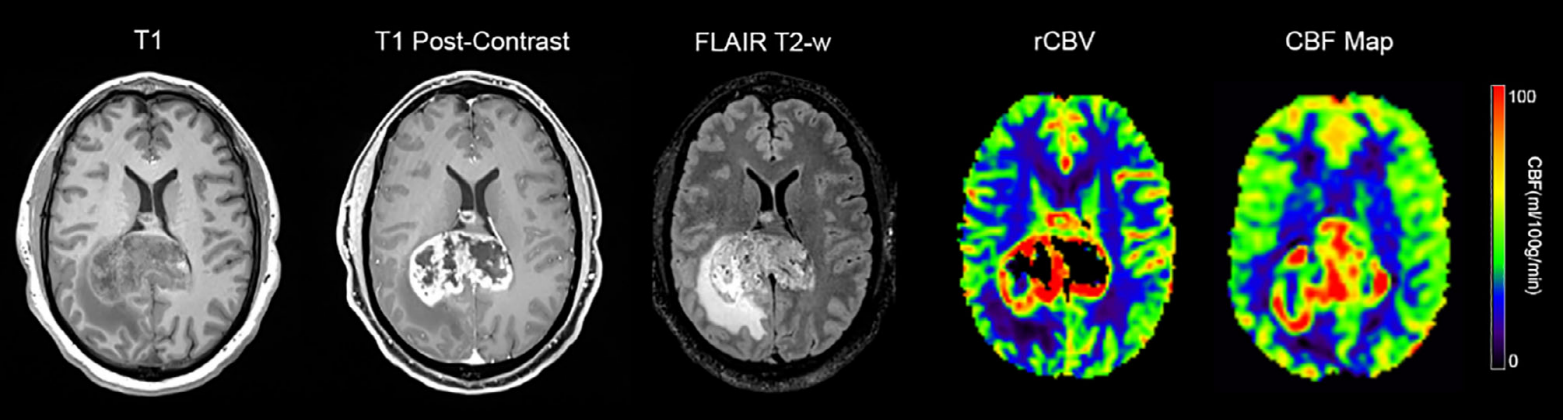
MRI results from a 48‐year‐old patient with a biopsy‐proven grade 4 glioblastoma. (Left‐to‐right) pre‐contrast T1‐weighted; post‐contrast T1‐weighted; T2‐weighted FLAIR images; relative cerebral blood volume (rCBV) map derived from dynamic susceptibility contrast (DSC) sequence; and cerebral blood flow (CBF) map derived from arterial spin labeling (ASL) are shown for a representative image slice. This example illustrates that the ASL CBF map is comparable to DSC rCBV imaging, showing high perfusion values at the periphery of the lesion. Note the partial volume effect on the central portion of the lesion on the CBF map that underestimates the necrotic area.

Although ASL images suffer from a lower signal‐to‐noise ratio than the DSC counterpart, a high correlation was found between ASL‐derived CBF and DSC‐measured rCBV in gliomas,^69^ with velocity‐selective ASL presenting even better results than normal pseudo‐continuous ASL.^70^ A comparison is shown in Fig. 4. The lack of expertise in reading ASL images by the radiologist is one of the major hurdles,^14^ and more training and tools, together with a more solid validation of ASL, are needed to demonstrate its ability to provide a valid alternative for DSC.

#### SUMMARY

Absolute measures of tumor blood flow can be obtained with ASL in the absence of exogenous intravenous contrast agents and the sequence is technically ready for clinical use. There is a correlation between ASL‐calculated CBF with tumor histology, grade, and microvascular density and no confusion with BBB leakage as in the post‐contrast T1‐weighted images. However, the added value to the conventional protocol and with respect to DSC still has to be proven in large multi‐center studies and diagnostic criteria need to be defined.

### Diffusion

#### OVERVIEW

Diffusion MRI is a technique based on motion‐sensitizing magnetic field gradients (*b*‐values), which attenuate the signal according to the motion direction and magnitude. Specifically, diffusion models are designed to mathematically estimate the attenuation that originates from the Brownian motion guided by the tissue's microstructure. The quantification of diffusion can then be used as a marker of pathology since the movement of water is dependent on parameters that affect the microstructure, such as cellularity, viscosity, or tortuosity of the extracellular space.

The most frequent method for measuring diffusion is the diffusion‐weighted EPI pulse sequence due to its speed and availability, but non‐EPI techniques (eg, turbo SE imaging or steady‐state free precession) can overcome some of the EPI limitations (eg, geometric warping in areas of susceptibility changes, such as bone/tissue interfaces).

The impedance of water molecule diffusion can be quantitatively assessed using the apparent diffusion coefficient (ADC) by eliminating the T2‐weighting that reduces the multi‐directional diffusivity at each point into a single number. In current clinical practice, diffusion tensor imaging (DTI) is also commonly used, enabling extraction of quantitative measures, such as fractional anisotropy (FA), or mean diffusivity (MD). FA is a measure of the dispersion of diffusion directionality, which is theoretically 0 in locations where water can freely diffuse in all directions and approaching 1 in highly anisotropic conditions where water diffuses along a single main axis (eg, in densely packed WM fibers). MD is mathematically similar to ADC and is a measure of the amount of diffusion in a given volume as an average of diffusion in all directions.

Besides DTI, less common diffusion‐MRI techniques include diffusion kurtosis imaging (DKI) and intravoxel incoherent motion (IVIM) imaging. In DKI, the deviation of diffusion from a Gaussian distribution can be quantified by kurtosis, a statistical measure that describes the non‐Gaussian behavior in biological tissues. The metrics most commonly extracted are mean kurtosis (MK, average of the diffusion kurtosis along all directions), axial kurtosis (kurtosis along the axial direction), and radial kurtosis (kurtosis along the radial direction). When higher *b*‐values are used (eg, *b* > 1500 s/mm^2^), kurtosis is more sensitive to microstructural environments and shorter molecular distances. IVIM imaging assumes a pure diffusion component and a pseudo‐diffusion component originating from the perfusion, and where multiple *b*‐values are used to obtain information on tissue microcirculation (perfusion) and microstructure (diffusion). The main objective of IVIM imaging is to disentangle the effects of perfusion and diffusion in MRI data to generate maps of true diffusion, pseudo‐diffusion coefficients, and of microvascular volume fractions.

#### CLINICAL APPLICATION

**FIGURE 5:**
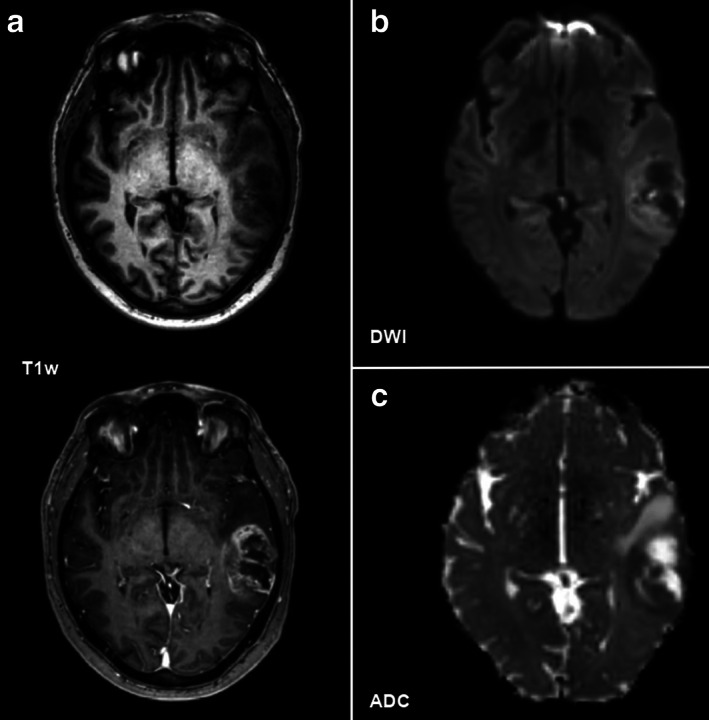
Diffusion restriction in a 45‐year‐old female patient with a left temporal high‐grade glioma (HGG) that showed contrast enhancement on T1‐weighted imaging (**a**; axial non‐contrast and contrast‐enhanced images). On diffusion‐weighted imaging (DWI, *b* = 1000 s/mm^2^), the borders of the lesion that primarily overlap with the contrast‐enhancing tumor parts show high signal intensity (**b**) that spatially corresponded to areas with a signal drop on apparent diffusion coefficient (ADC) maps (**c**), indicating restricted diffusion most probably due to focal high cellularity of the glioma.

Regarding the preoperative quantitative assessment of glioma by diffusion MRI, ADC values could reflect tumor cellularity and the tumor burden (Fig. 5). Specifically, ADC values were successfully applied to differentiate between LGG and HGG, with a summarized sensitivity and specificity of 0.85 and 0.80, respectively, and a summary ROC‐based AUC of 0.90 as derived from a meta‐analysis consisting of 15 studies.^71^ Also, ADC values were significantly higher in IDH1‐mutated gliomas than in IDH1‐wild‐type gliomas.^72^ Furthermore, ADC was associated with different histologic and genetic types of WHO grade II‐III diffuse gliomas, considering both the 2007 and 2016 WHO classifications, especially for 1p/19q co‐deletion and IDH1 mutation status.^73^ ADC values were also successfully used in a multi‐center study to predict the EGFR amplification status of IDH‐wild‐type WHO grade II‐III gliomas.^74^ Specifically, lower mean ADC and the lower 5th percentile of ADC values were potentially useful imaging biomarkers for EGFR amplification in IDH‐wild‐type glioma.^74^ Previously, the assessment of ADC values in different parenchyma and brain tumor regions has also been considered, and for patients with IDH‐mutated tumors, ADC values were significantly higher in tumor tissue than in marginal areas of the tumor, while there were no significant differences in terms of ADC values between tumor tissue and marginal areas of the tumor for patients with IDH‐wild‐type tumors.^75^ The assessment of ADC values in H3 K27M histone‐mutant diffuse midline glioma, a recently classified neoplasm, revealed that mean ADC was 0.84 ± 0.15 × 10^−3^ mm^2^/s.^76^ Another study also addressed the diffuse midline glioma H3 K27‐mutant, and concluded that lower ADC values in non‐enhancing areas may be related to the normal expression of ATRX.^77^

It has been demonstrated that maximal and/or mean FA values are usually significantly higher in HGG, which may be due not only to infiltration, but also to disruption of fibers compared to LGG with mainly infiltration without disruption.^78^ Yet, the opposite has been reported for WHO grade II and grade III gliomas as well, which was mostly because FA values were dominated by tumor infiltration that caused disruption of the neuronal, axonal, and glial microstructure rather than by changes due to abnormal cellularity.^79^ In addition to differentiating glioma with respect to the WHO grading, studies have investigated the ability to discriminate between tumors according to IDH mutation status, and maximal FA and the ratio of maximal FA (maximal FA divided by the contralateral normal FA) were significantly different between oligodendroglial tumors with IDH mutations and those without mutations, yielding areas under the curve (AUCs) of 0.79 and 0.82, respectively.^80^ Moreover, a positive association was observed between IDH1 status and MD in a cohort of patients with HGG.^81^ Similarly, whole‐tumor histograms, as well as texture analysis of FA maps, enabled prediction of the IDH1‐mutation and 1p/19q‐codeletion status in patients with WHO grade II gliomas.^82^ In addition, evaluation of FA in the peritumoral region may enable conclusions to be drawn at baseline about potential spatial recurrence patterns.^83^

More advanced techniques, such as DKI and IVIM imaging, have been applied to preoperative imaging in neuro‐oncology. Specifically, MK values derived from DKI were used for grading, and it has been shown that MK values increased with higher glioma malignancy.^84^ Furthermore, it has been demonstrated that axial kurtosis and radial kurtosis values were also increased in cases of HGG vs. LGG, outlining the application of MK for grading (WHO grades II‐IV) and predicting Ki‐67 as a metric related to cellularity.^85^ Moreover, another metric used was MK, a parameter derived from fast kurtosis imaging, which showed increased values in HGG compared to LGG.^86^ Another possible application of DKI in the setting of integrated glioma diagnosis is the prediction of the IDH mutation status based on the intrinsic tumoral heterogeneity as expressed by variant intravoxel kurtosis features.^87^ Similarly, IVIM imaging has shown great potential, since it has demonstrated the ability to differentiate between HGG and LGG, and to support the prediction of survival for newly diagnosed HGG and the treatment outcome in the course of therapy.^88^, ^89^

#### VALIDATION

The application of diffusion MRI in neuro‐oncological imaging may represent a promising technique to assess tissue microstructure in the presence of glioma. However, there are multiple sequences and analysis techniques available that may not be feasible for each center and MRI system used. One shortcoming is that most centers use a single‐shot EPI method for diffusion sequences because of its short acquisition times. However, this approach provides limited spatial resolution and can be adversely affected by susceptibility artifacts that can mislead qualitative and quantitative analysis, notably at tissue interfaces. Moreover, EPI‐based eddy currents can create image distortions that confound diffusion parameter evaluation. Furthermore, motion artifacts are a relevant problem for sequences with long acquisition times, including diffusion MRI.

While basic diffusion‐weighted imaging (DWI) is usually acquired rapidly, multi‐directional DTI acquisitions can take several minutes and therefore are particularly prone to head motion artifacts. Several processing tools may be used to correct residual motion artifacts in DTI results, and a combined approach using prospective and retrospective motion‐correction techniques could facilitate overall distortion correction. In particular, DWI is also prone to perfusion effects from water movement in the capillary network, which can possibly overestimate ADC in highly vascular tumors. Conversely, diffusion and perfusion effects can be separated and specifically used for glioma imaging with the IVIM technique.

#### SUMMARY

For preoperative imaging, DWI is among the most frequently used sequences besides conventional structural imaging. Particularly, basic measurements (i.e., ADC and FA) have been investigated in detail for the differentiation between certain glioma entities and, more recently, also to predict different glioma molecular subtypes. However, mostly EPI‐based and DTI techniques have been applied, while more advanced methods such as DKI or IVIM imaging have become increasingly available for the clinical setting and can provide more detailed insights into tissue microstructure.

### Vessel Imaging

#### OVERVIEW

##### MR Angiography

The principal method used for MRA is time‐of‐flight (TOF) angiography, also known as inflow angiography, which does not require GBCA administration, but instead visualizes arteries using the magnetically unsaturated spins of flowing blood, which has more signal than the surrounding tissue.

##### Vessel‐Architectural Imaging

**FIGURE 6:**
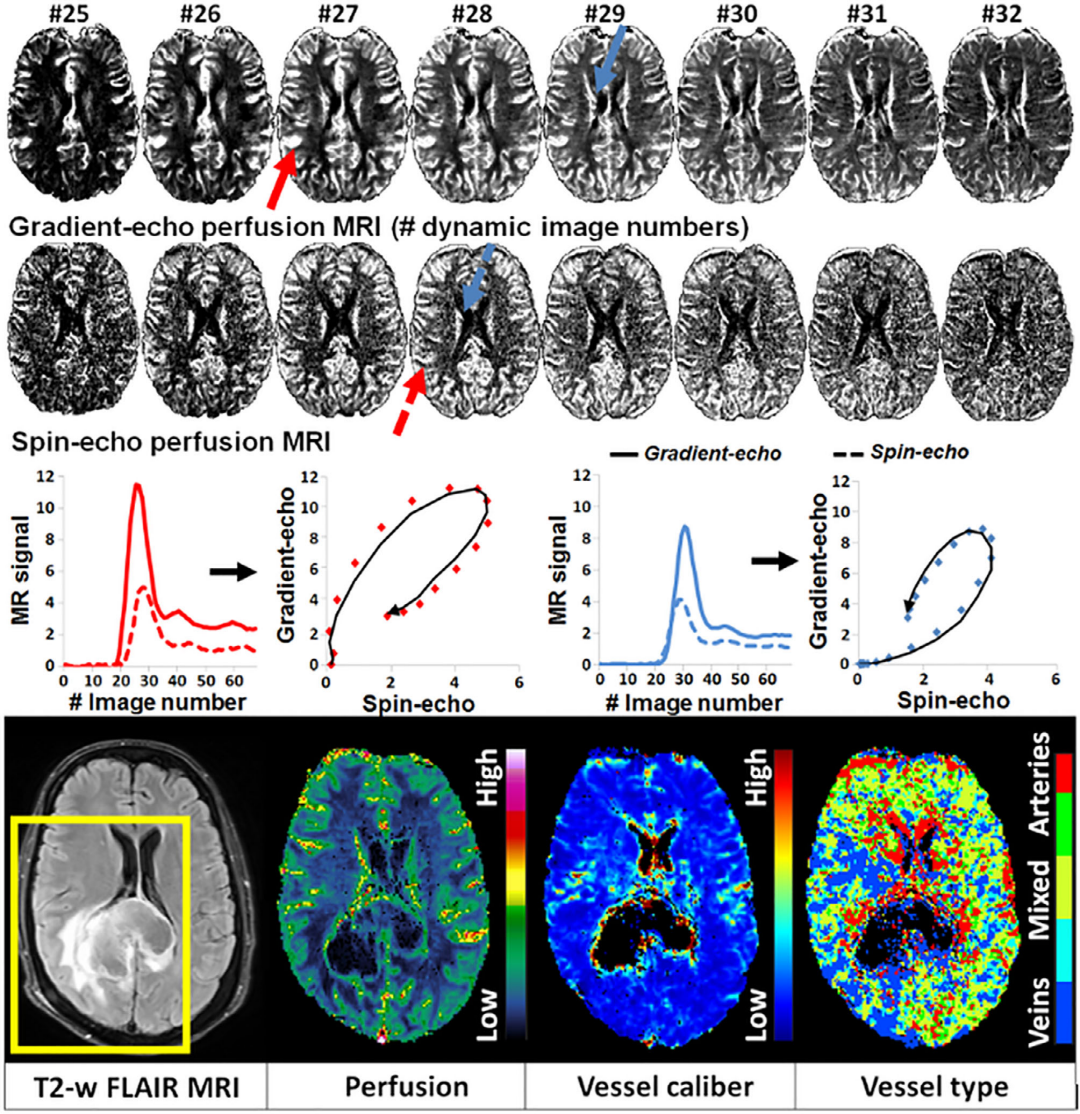
Perfusion MRI and vessel‐architectural imaging (VAI). Merging two perfusion readouts (gradient‐ and spin‐echo MRI) creates unique signal curve “loops” that scale with vessel calibers (slope) and vessel type (loop direction). Note the impaired, venous‐like dominance of the peri‐tumoral area (yellow box) on the vessel type map of a glioblastoma not observed on other images. Adapted from Emblem et al, Nature Medicine 2003.^92^

By acquiring a combined GRE and SE EPI sequence during an intravenous GBCA injection, the average vessel calibers can be estimated in glioma by so‐called vessel‐architectural imaging (VAI), sometimes also referred to as vessel size imaging.^90^ The highly susceptibility‐sensitive GRE signal is sensitive to blood vessels of all calibers, whereas SE is predominantly sensitive to microscopic vessels or capillaries (radius < 10 μm).^91^ This phenomenon is further exploited in VAI to provide further insight into vessel size and type (Fig. 6). The differing sensitivity to the susceptibility effect of SE and gradient‐echo readouts creates a relative and temporal shift in the shape and peak position of the GBCA‐induced signal curves when plotted.^92^

#### CLINICAL APPLICATION

##### MR Angiography

Limited studies have reported attempts to assess glioma based on MRA techniques in humans. These are small pilot or older studies, none of which includes histological differentiation.

In line with works by Kadota et al that demonstrated the value of TOF to identify pathological neovascularization,^93^ Radbruch et al presented a limited 7T pilot, which showed that high‐resolution (0.3 × 0.3 × 0.4 mm) TOF made arterial vessels not only visible, but also quantifiable, with more and denser vessels in glioblastoma compared to normal tissue.^94^ A more recent study found that a new analysis method that measures local vessel orientation angles can help differentiate normal from abnormal tumor vessels.^95^

MRA studies are especially driven by their utility for neurosurgical planning, eg, to delineate lenticulostriate arteries. One smaller study showed that hypervascularization of glioblastoma with contrast‐enhanced MR angiography (CE‐MRA)^96^ is an excellent marker of reduced OS.

##### Vessel‐Architectural Imaging

Several VAI studies with a medium‐sized patient group (50–100 participants) have demonstrated successful differentiation by brain tumor histology in patients with glioma. VAI was successfully applied to examine differences in vasculature between untreated glioma types according to the 2016 WHO classification.^97^ This also included differentiation by IDH mutation based on VAI parameters at an AUC of 0.782.^98^ Notably, no studies exist using the WHO 2021 classification. Moreover, VAI‐associated biomarkers are valuable in the assessment of the heterogeneity of the glioma microvasculature that arises from the angiogenic activity of these tumors. VAI maps proved helpful for the delineation of glioma from peripheral edema.^99^ Stadlbauer et al suggested using vascular‐induced peak shift and the microvessel type indicator to allow early neovascularization detection, while curvature might be a marker for the severity of vasogenic edema.^100^

#### VALIDATION

##### MR Angiography

Different studies have suggested that MR angiography has substituted conventional angiography for the assessment of intracranial vessels.^101^ TOF angiography (i.e., the main MR angiography technique), when used on clinical scanners (≤3T), is less accurate than contrast‐enhanced MRA (CEMRA) and digital subtraction angiography (DSA).^102^ However, DSA and CEMRA are invasive, or, at best, require contrast agents that trigger adverse effects. Ultra‐high magnetic field (i.e., 7T) scanners have opened the door to a substantial improvement in the quality and contrast of the structures of interest in TOF angiography. Nevertheless, these advances are far from clinical application due to the limited availability of 7T scanners and artifacts due to magnetic field inhomogeneity.

##### Vessel‐Architectural Imaging

Outside a few major research institutions, VAI is not yet clinically available for patients with glioma, even though both vessel size and perfusion parameters could be derived simultaneously from the same dataset. This is mainly due to a lack of standardization of optimal imaging parameters and intricate post‐processing routines requiring dedicated analysis software, and special expertise. Finally, based on the complex biology and overlapping distribution of tumor‐vessel calibers across different brain tumor types, quantitative assessments using a cut‐off value for vessel size may not discriminate between tumor types or treatment outcomes. Instead, relative measures of vessel size are most often used, which further increases the complexity of the technique. Taken together, to date, only a relatively small number of clinical studies have been performed and with limited (N < 50) and diverse patient cohorts.

#### SUMMARY

##### MR Angiography

TOF angiography, the most commonly used method for MR angiography, has advantages over other established conventional angiography modalities, such as CEMRA or DSA, as it is noninvasive and does not require external contrast agents. However, on clinical scanners, TOF angiography lacks the accuracy of conventional modalities, and its use in glioma analysis is currently limited to very specific studies mainly related to surgical planning or analysis of the vascular architecture from a morphological point of view.

##### Vessel‐Architectural Imaging

Because angiogenesis is a hallmark of cancer, assessment of the vasculature by VAI constitutes an attractive tool for diagnosis and monitoring. However, the technique still suffers from a lack of clinical validation by cross‐vendor repeatability and reliability tests. With a continued focus on implementation guidelines and automatic analysis pipelines, the utility and availability of VAI may greatly improve even outside the research community.

### Relaxometry and MR Fingerprinting (MRF)

#### OVERVIEW

Quantitative mapping of T1 and T2 relaxation times offers more specific and robust characterization of glioma compared to conventional structural imaging techniques. Early pulse sequences involved changing one acquisition parameter at a time and, while inversion recovery mapping of T1 and SE mapping of T2 remain gold‐standard techniques, they are prohibitively time‐consuming for clinical use.^103^ Acquisition times can be greatly reduced using simultaneous multiparametric mapping techniques, such as Magnetic Resonance Image Compilation (MAGiC),^104^ and Magnetic Resonance Fingerprinting (MRF).^105^

**FIGURE 7:**
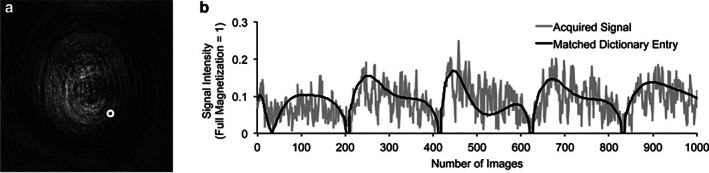
Schematic depicting an example of a fingerprint (**a**) with its corresponding best‐acquired signal and matched dictionary entry (**b**). Reproduced with permission from Jiang et al Magn Reson Med 2015.^106^

In MRF, a pulse sequence with pseudo‐randomly varying acquisition parameters (eg, flip angle and repetition time) results in temporally varying measured signals, termed fingerprints, for each voxel. Fingerprints are dictated by multiple relaxation mechanisms (eg, T1, T2), and a dictionary can be generated by pre‐calculating predicted combinations of parameter values using simulations and Bloch equations. The acquired fingerprints are matched with the dictionary entries to deduce voxel‐wise parameter values and reconstruct quantitative maps (Fig. 7).

#### CLINICAL APPLICATION

##### Biological Relevance of T1 and T2 Mapping

T1 and T2 relaxation times of tissue are influenced by the number of lipids, proteins, macromolecules, and paramagnetic materials present, as well as the local water content, microstructure, and cellularity. The T1 and T2 relaxation times of tumors have been found to be longer than that of surrounding healthy tissue,^107^, ^108^, ^109^ with contrast thought to be mainly driven by increased hydration in the form of intra‐tumoral edema,^110^ which occurs due to leaky vessels as a consequence of neovascularization.

Further classification or grading of gliomas has been investigated but is less straightforward. In a 2D fingerprinting study by Badve et al, solid tumor regions of LGG and metastases were significantly different, but overlap in the range between tissues meant it was not possible to distinguish between LGG and HGG based on average relaxation times.^107^

That study concluded that relaxation times in glioma were mainly driven by cellularity type and the common glial origin, meaning tumor grades could not be differentiated based on mean values alone. Further radiomics analysis of the same quantitative fingerprinting dataset revealed significant differences between metastasis, LGGs, and HGGs.^111^ A significant correlation has also been found between lower T1 entropy (representing lower heterogeneity) and survival.^111^ In a small study including 14 children and 9 young adults (ages 1–34), significant differences were found in mean MRF‐derived T1/T2 values between 19 LGGs and 4 HGGs, and between tumor and contralateral white matter.^108^

**FIGURE 8:**
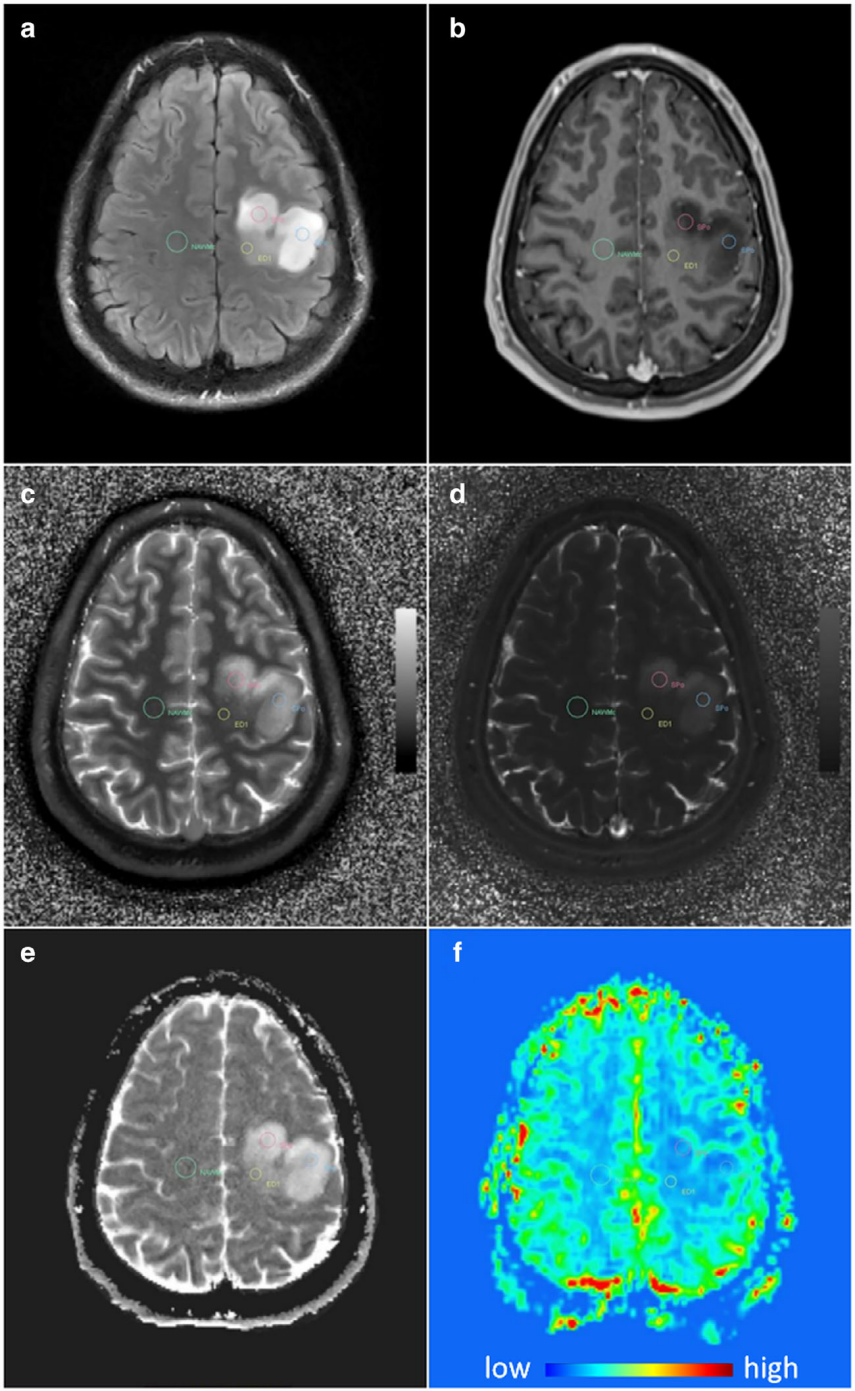
Quantitative maps derived from MRF in an astrocytoma, IDH‐mutant, show increased T1 and T2 values within the tumor mass. The MRF T1 map (**c**) and T2 map (**d**) are compared to FLAIR (**a**), T1w contrast‐enhanced MRI (**b**), an ADC map (**e**), and perfusion‐weighted imaging (**f**). Reproduced from Springer et al 2022 under CC license.^112^

More recent studies have addressed the role of multi‐echo SE T2 mapping in IDH mutation status of adult LGGs and showed an increase in T2 in IDH‐mutant glioma compared to IDH‐wildtype glioma.^109^ This was also observed in an MRF study, which showed an increase in T1 values in the solid component of IDH mutant glioma, compared to wildtype.^112^ An example of these findings is displayed in Fig. 8.

Quantitative imaging could also provide insight into the extent of non‐enhancing tumor invasion into peritumoral edema. Quantitative MAGiC mapping found T1 and T2 relaxation times could detect tumor invasion and the peritumoral regions of T1 maps showed post‐contrast enhancement, neither of which was visible on conventional imaging.^113^, ^114^ Variable flip angle T1 mapping and multi‐echo SE T2 mapping have yielded similar results,^115^ suggesting a broader infiltration can be identified using postcontrast quantitative mapping. Edema surrounding LGGs was found to have lower mean T1 times mapped by MRF than the edema around HGGs,^107^ but postcontrast MRF studies have not yet been reported.

#### VALIDATION

MRF is a recent technique that holds great promise in introducing rapid quantitative T1 and T2 mapping into the clinic; however, only a small number of clinical studies have been performed. These studies have had limited patient cohort numbers, with an uneven distribution of glioma grades and types, as well as different methods for tumor segmentation and data analyses. The majority of MRF studies have been implemented using a 2D sequence. However, a proof‐of‐concept study evaluated a 3D fingerprinting‐style, quantitative, transient‐state imaging (QTI) sequence in nine glioma patients (grades 2–4) with varying treatment histories, and showed the feasibility of 3D acquisitions in glioma, with a whole‐brain acquisition time under 7 minutes.^116^ Finally, the repeatability and reliability of MRF in a clinical environment need to be further validated to confirm the positive results in healthy brains.^117^

#### SUMMARY

Still in its infancy, MRF promises to be a technique that will aid in the characterization and delineation of brain tumors, making quantitative T1 and T2 imaging acquisitions rapid and ready for use in a clinical environment.^118^ Larger validation studies are expected in the near future as MRF becomes more widely available.

### Overview: Level of Clinical Validation

## Discussion

In this review, a working group of the GliMR COST action has summarized the evidence for clinical use of advanced MRI for preoperative glioma characterization.

The most frequently used advanced‐MRI sequence is DWI. The likely reasons—the ease of implementation, wide availability, and frequent use in other pathologies—might be more important for the widespread use than DWI's diagnostic potential. Notably, advanced diffusion sequences, like IVIM and DKI, are used only marginally in glioma due to their lower availability and more difficult processing and interpretation.

In contrast, DSC is an exemplary case of accelerating the clinical translation of advanced MRI. DSC involves contrast‐agent injection, input function delineation, tracer‐kinetic modeling, and value normalization. Despite its complexity, DSC is commonly used in glioma imaging^14^ owing mainly to the extensive work invested in DSC validation. This work culminated recently by introducing consensus recommendations,^18^ which provide clear instructions on the measurement and evaluation processes and is backed up by robust validation. Such recommendations constitute an important step toward clinical acceptance and are missing for nearly all other advanced MRI techniques.

The level of clinical validation also depends on more general determinants. Glioma is a relatively rare disease, making it difficult to collect enough data for statistically robust assessments. The Brain Tumor Segmentation (BraTS) repository initiative partly addresses this by collecting multi‐institutional pre‐operative multi‐parametric MRI scans of patients with glioma.^119^ Still, regardless of the level of good‐quality evidence, lack of widespread use may boil down to something as simple as limited demand and early adoption from key practitioners due to preference, education, and above all awareness. With increasing demand, shorter time to read exams, and reduced hospital budgets, it may be difficult for the radiologist or end user to stay up to date with the wide range of available methods, or find the time and resources to lead the clinical implementation. For any new technique, adaptation and priority of use will be weighed against the cost and resources of the exam, its associated clinical workup, and the initial implementation and training of bioengineers and neuroradiologists. In practice, this may be a question of healthcare reimbursement policies and insurance coverage, which usually do not include techniques that are not part of the guidelines. Several initiatives are trying to make up for this difference by reviewing the abilities of advanced MRI^6^; building networks of professionals in glioma imaging, providing educational resources and processing tools for advanced MRI, and connecting with other professionals in neuro‐oncology as in GliMR^10^; working on providing open source software for data analysis like the Open Science Initiative For Perfusion Imaging (OSIPI)^120^; or seeking to improve the practical value of quantitative imaging biomarkers as the Quantitative Imaging Biomarkers Alliances (QIBA) does.^121^

Despite the promise of improved diagnostic efficiency of the new imaging biomarkers, clinical translation will need to respect the cost–benefit ratio of its use and the patient's health status. This translates to keeping the acquisition duration mostly unchanged and replacing old sequences with newer ones only if the added value compensates for the hurdles associated with introducing new techniques. For example, while DCE measures both the permeability of the BBB and vascularization and is potentially more useful than DSC, it is not straightforward to measure both sequences within a single session. Therefore, DSC is currently prioritized as a quicker and more robust technique that already has well‐established guidelines and a much higher level of validation. Overall, GBCA use in gliomas is likely to be reduced in the future due to added burden to costs, logistics, and patient discomfort burdens, as well as safety issues raised by both American and European pharmacological safety agencies. ASL,^57^ BBB‐ASL,^122^ and machine‐learning‐based techniques are on a good path to complement and maybe eventually replace DSC, DCE, and post‐contrast T1‐weighted scans, respectively, in many glioma patients and especially children. Although MRF has not been validated, its promise to provide fully quantitative measurements of relaxation times could obviate the conventional protocol and thus make space for other advanced sequences like VSI, CEST, or MRS.

Even when advanced diagnostic tools are implemented and available to the end user, their clinical use is challenged by an inherent paradox. As imaging techniques become more advanced, so do their resulting imaging biomarkers, where any metric will move away from a simple binary (yes/no) or cutoff (above/below) value for characterization. With a higher‐level technique, the complex biology and function of cancer will arguably be assessed in a more accurate and unique way, but at the cost of more difficult interpretations. Multi‐parametric assessment combining several advanced MRI techniques may also further improve glioma characterization and reduce the bias of any single technique toward certain biological or functional properties of the tissue. However, a multi‐parametric approach will also add to the complexity of the analysis. As a result, in a busy practice there may be no time, nor may it be technically feasible, for radiologists to process this data. For advanced imaging methods to be widely adopted, a strong focus is required on translating clinically ready technology into commercial software directly embedded in the hospital‐wide picture archiving and communication system (PACS). This will allow for standardization and start‐to‐end automatic pipelines as an alternative to laborious and user‐dependent alternatives.

In conclusion, effective treatment of gliomas is still an unmet clinical need that is, in part, reflected by their wide‐ranging intra‐ and inter‐individual biological heterogeneity. Targeted therapy, which has demonstrated promising results in other cancers, has largely failed in gliomas. To address this challenge, the advanced imaging techniques discussed in this review hold the potential to support better clinical decisions for tumor characterization and subsequent treatment. By focusing on what these techniques are currently missing to advance their clinical readiness level, the imaging community can help make advanced MRI for glioma diagnosis and therapy clinically available, personalized, and effective.

## Supporting information


**Appendix S1.** Supplementary Information
