# Supplementary material for: Advanced MR Techniques for Preoperative Glioma Characterization: Part 1
Source: J Magn Reson Imaging. 2023 Mar 3;57(6):1655–75. doi: 10.1002/jmri.28662 (PMC10946498; doi:10.1002/jmri.28662)
Supplement: Supplementary file 1 — Appendix S1. Supplementary Information [file JMRI-57-1655-s001.docx]

# Supplementary materials - part 1

## References Level of Validation Table - DSC

Boxerman, Jerrold L., Chad C. Quarles, Leland S. Hu, Bradley J. Erickson, Elizabeth R. Gerstner, Marion Smits, Timothy J. Kaufmann, et al. 2020. “Consensus Recommendations for a Dynamic Susceptibility Contrast MRI Protocol for Use in High-Grade Gliomas.” Neuro-Oncology 22 (9): 1262–75.

Connelly, Jennifer M., Melissa A. Prah, Fernando Santos-Pinheiro, Wade Mueller, Elizabeth Cochran, and Kathleen M. Schmainda. 2021. “Magnetic Resonance Imaging Mapping of Brain Tumor Burden: Clinical Implications for Neurosurgical Management: Case Report.” Neurosurgery Open 2 (4): okab029.

Donahue, K. M., H. G. Krouwer, S. D. Rand, A. P. Pathak, C. S. Marszalkowski, S. C. Censky, and R. W. Prost. 2000. “Utility of Simultaneously Acquired Gradient-Echo and Spin-Echo Cerebral Blood Volume and Morphology Maps in Brain Tumor Patients.” Magnetic Resonance in Medicine: Official Journal of the Society of Magnetic Resonance in Medicine / Society of Magnetic Resonance in Medicine 43 (6): 845–53.

Gerstner, Elizabeth R., Zheng Zhang, James R. Fink, Mark Muzi, Lucy Hanna, Erin Greco, Melissa Prah, et al. 2016. “ACRIN 6684: Assessment of Tumor Hypoxia in Newly Diagnosed Glioblastoma Using 18F-FMISO PET and MRI.” Clinical Cancer Research: An Official Journal of the American Association for Cancer Research 22 (20): 5079–86.

Henriksen, Otto M., María Del Mar Álvarez-Torres, Patricia Figueiredo, Gilbert Hangel, Vera C. Keil, Ruben E. Nechifor, Frank Riemer, et al. 2022. “High-Grade Glioma Treatment Response Monitoring Biomarkers: A Position Statement on the Evidence Supporting the Use of Advanced MRI Techniques in the Clinic, and the Latest Bench-to-Bedside Developments. Part 1: Perfusion and Diffusion Techniques.” Frontiers in Oncology 12 (March): 810263.

Hu, Leland S., Jennifer M. Eschbacher, Joseph E. Heiserman, Amylou C. Dueck, William R. Shapiro, Seban Liu, John P. Karis, et al. 2012. “Reevaluating the Imaging Definition of Tumor Progression: Perfusion MRI Quantifies Recurrent Glioblastoma Tumor Fraction, Pseudoprogression, and Radiation Necrosis to Predict Survival.” Neuro-Oncology 14 (7): 919–30.

Hu, L. S., Z. Kelm, P. Korfiatis, A. C. Dueck, C. Elrod, B. M. Ellingson, T. J. Kaufmann, et al. 2015. “Impact of Software Modeling on the Accuracy of Perfusion MRI in Glioma.” AJNR. American Journal of Neuroradiology 36 (12): 2242–49.

Jafari-Khouzani, Kourosh, Kyrre E. Emblem, Jayashree Kalpathy-Cramer, Atle Bjørnerud, Mark G. Vangel, Elizabeth R. Gerstner, Kathleen M. Schmainda, et al. 2015. “Repeatability of Cerebral Perfusion Using Dynamic Susceptibility Contrast MRI in Glioblastoma Patients.” Translational Oncology 8 (3): 137–46.

Juan-Albarracín, Javier, Elies Fuster-Garcia, Alexandre Pérez-Girbés, Fernando Aparici-Robles, Ángel Alberich-Bayarri, Antonio Revert-Ventura, Luis Martí-Bonmatí, and Juan M. García-Gómez. 2018. “Glioblastoma: Vascular Habitats Detected at Preoperative Dynamic Susceptibility-Weighted Contrast-Enhanced Perfusion MR Imaging Predict Survival.” Radiology 287 (3): 944–54.

Korfiatis, Panagiotis, Timothy L. Kline, Zachary S. Kelm, Rickey E. Carter, Leland S. Hu, and Bradley J. Erickson. 2016. “Dynamic Susceptibility Contrast-MRI Quantification Software Tool: Development and Evaluation.” Tomography (Ann Arbor, Mich.) 2 (4): 448–56.

Lev, Michael H., Yelda Ozsunar, John W. Henson, Amjad A. Rasheed, Glenn D. Barest, Griffith R. Harsh 4th, Markus M. Fitzek, et al. 2004. “Glial Tumor Grading and Outcome Prediction Using Dynamic Spin-Echo MR Susceptibility Mapping Compared with Conventional Contrast-Enhanced MR: Confounding Effect of Elevated rCBV of Oligodendrogliomas [corrected].” AJNR. American Journal of Neuroradiology 25 (2): 214–21.

Maeda, M., S. Itoh, H. Kimura, T. Iwasaki, N. Hayashi, K. Yamamoto, Y. Ishii, and T. Kubota. 1993. “Tumor Vascularity in the Brain: Evaluation with Dynamic Susceptibility-Contrast MR Imaging.” Radiology 189 (1): 233–38.

McCullough, Brendan J., Valerie Ader, Brian Aguedan, Xu Feng, Daniel Susanto, Tara L. Benkers, John W. Henson, et al. 2018. “Preoperative Relative Cerebral Blood Volume Analysis in Gliomas Predicts Survival and Mitigates Risk of Biopsy Sampling Error.” Journal of Neuro-Oncology 136 (1): 181–88.

Milchenko, Mikhail V., Dhanashree Rajderkar, Pamela LaMontagne, Parinaz Massoumzadeh, Ronald Bogdasarian, Gordon Schweitzer, Tammie Benzinger, Dan Marcus, Joshua S. Shimony, and Sarah Jost Fouke. 2014. “Comparison of Perfusion- and Diffusion-Weighted Imaging Parameters in Brain Tumor Studies Processed Using Different Software Platforms.” Academic Radiology 21 (10): 1294–1303.

Patel, Praneil, Hediyeh Baradaran, Diana Delgado, Gulce Askin, Paul Christos, Apostolos John Tsiouris, and Ajay Gupta. 2017. “MR Perfusion-Weighted Imaging in the Evaluation of High-Grade Gliomas after Treatment: A Systematic Review and Meta-Analysis.” Neuro-Oncology 19 (1): 118–27.

Prah, M. A., S. M. Stufflebeam, E. S. Paulson, J. Kalpathy-Cramer, E. R. Gerstner, T. T. Batchelor, D. P. Barboriak, B. R. Rosen, and K. M. Schmainda. 2015. “Repeatability of Standardized and Normalized Relative CBV in Patients with Newly Diagnosed Glioblastoma.” AJNR. American Journal of Neuroradiology 36 (9): 1654–61.

Prah, Melissa A., Mona M. Al-Gizawiy, Wade M. Mueller, Elizabeth J. Cochran, Raymond G. Hoffmann, Jennifer M. Connelly, and Kathleen M. Schmainda. 2018. “Spatial Discrimination of Glioblastoma and Treatment Effect with Histologically-Validated Perfusion and Diffusion Magnetic Resonance Imaging Metrics.” Journal of Neuro-Oncology 136 (1): 13–21.

Schmainda, Kathleen M., Melissa A. Prah, Helga Marques, Eunhee Kim, Daniel P. Barboriak, and Jerrold L. Boxerman. 2021. “Value of Dynamic Contrast Perfusion MRI to Predict Early Response to Bevacizumab in Newly Diagnosed Glioblastoma: Results from ACRIN 6686 Multicenter Trial.” Neuro-Oncology 23 (2): 314–23.

Schmainda, Kathleen M., Scott D. Rand, Allen M. Joseph, Rebecca Lund, B. Doug Ward, Arvind P. Pathak, John L. Ulmer, Michael A. Badruddoja, and Hendrikus G. J. Krouwer. 2004. “Characterization of a First-Pass Gradient-Echo Spin-Echo Method to Predict Brain Tumor Grade and Angiogenesis.” AJNR. American Journal of Neuroradiology 25 (9): 1524–32.

Schmainda, Kathleen M., Zheng Zhang, Melissa Prah, Bradley S. Snyder, Mark R. Gilbert, A. Gregory Sorensen, Daniel P. Barboriak, and Jerrold L. Boxerman. 2015. “Dynamic Susceptibility Contrast MRI Measures of Relative Cerebral Blood Volume as a Prognostic Marker for Overall Survival in Recurrent Glioblastoma: Results from the ACRIN 6677/RTOG 0625 Multicenter Trial.” Neuro-Oncology 17 (8): 1148–56.

Schmainda, K. M., M. A. Prah, S. D. Rand, Y. Liu, B. Logan, M. Muzi, S. D. Rane, et al. 2018. “Multisite Concordance of DSC-MRI Analysis for Brain Tumors: Results of a National Cancer Institute Quantitative Imaging Network Collaborative Project.” AJNR. American Journal of Neuroradiology 39 (6): 1008–16.

Soliman, Radwa K., Sara A. Gamal, Abdel-Hakeem A. Essa, and Mostafa H. Othman. 2018. “Preoperative Grading of Glioma Using Dynamic Susceptibility Contrast MRI: Relative Cerebral Blood Volume Analysis of Intra-Tumoural and Peri-Tumoural Tissue.” Clinical Neurology and Neurosurgery 167 (April): 86–92.

Stokes, Ashley M., Jack T. Skinner, and C. Chad Quarles. 2014. “Assessment of a Combined Spin- and Gradient-Echo (SAGE) DSC-MRI Method for Preclinical Neuroimaging.” Magnetic Resonance Imaging 32 (10): 1181–90.

Sugahara, T., Y. Korogi, M. Kochi, I. Ikushima, T. Hirai, T. Okuda, Y. Shigematsu, et al. 1998. “Correlation of MR Imaging-Determined Cerebral Blood Volume Maps with Histologic and Angiographic Determination of Vascularity of Gliomas.” AJR. American Journal of Roentgenology 171 (6): 1479–86.

## References Level of Validation Table - DCE

Anzalone, Nicoletta, Antonella Castellano, Marcello Cadioli, Gian Marco Conte, Valeria Cuccarini, Alberto Bizzi, Marco Grimaldi, et al. 2018. “Brain Gliomas: Multicenter Standardized Assessment of Dynamic Contrast-Enhanced and Dynamic Susceptibility Contrast MR Images.” Radiology 287 (3): 933–43.

Artzi, Moran, Gilad Liberman, Deborah T. Blumenthal, Felix Bokstein, Orna Aizenstein, and Dafna Ben Bashat. 2018. “Repeatability of Dynamic Contrast Enhanced vp Parameter in Healthy Subjects and Patients with Brain Tumors.” Journal of Neuro-Oncology 140 (3): 727–37.

Liang, Jianye, Dexiang Liu, Peng Gao, Dong Zhang, Hanwei Chen, Changzheng Shi, and Liangping Luo. 2018. “Diagnostic Values of DCE-MRI and DSC-MRI for Differentiation Between High-Grade and Low-Grade Gliomas: A Comprehensive Meta-Analysis.” Academic Radiology 25 (3): 338–48.

Petralia, Giuseppe, Paul E. Summers, Andrea Agostini, Roberta Ambrosini, Roberta Cianci, Giulia Cristel, Linda Calistri, and Stefano Colagrande. 2020. “Dynamic Contrast-Enhanced MRI in Oncology: How We Do It.” La Radiologia Medica 125 (12): 1288–1300.

## References Level of Validation Table - ASL

Alsaedi, Amirah, Fabio Doniselli, Hans Rolf Jäger, Jasmina Panovska-Griffiths, Antonio Rojas-Garcia, Xavier Golay, and Sotirios Bisdas. 2019. “The Value of Arterial Spin Labelling in Adults Glioma Grading: Systematic Review and Meta-Analysis.” Oncotarget 10 (16): 1589–1601.

Alsaedi, Amirah Faisal, David Lee Thomas, Enrico De Vita, Jasmina Panovska-Griffiths, Sotirios Bisdas, and Xavier Golay. 2022. “Repeatability of Perfusion Measurements in Adult Gliomas Using Pulsed and Pseudo-Continuous Arterial Spin Labelling MRI.” Magma 35 (1): 113–25.

Baas, K. P. A., J. Petr, J. P. A. Kuijer, A. J. Nederveen, H. J. M. M. Mutsaerts, and K. C. C. van de Ven. 2021. “Effects of Acquisition Parameter Modifications and Field Strength on the Reproducibility of Brain Perfusion Measurements Using Arterial Spin-Labeling.” AJNR. American Journal of Neuroradiology 42 (1): 109–15.

Chen, Yufen, Danny J. J. Wang, and John A. Detre. 2011. “Test-retest reliability of arterial spin labeling with common labeling strategies.” Journal of Magnetic Resonance Imaging: JMRI 33 (1522-2586 (Electronic)): 940–49.

Dangouloff-Ros, Volodia, Christophe Deroulers, Frantz Foissac, Mathilde Badoual, Eimad Shotar, David Grévent, Raphael Calmon, et al. 2016. “Arterial Spin Labeling to Predict Brain Tumor Grading in Children: Correlations between Histopathologic Vascular Density and Perfusion MR Imaging.” Radiology 281 (2): 553–66.

Falk Delgado, Alberto, Francesca De Luca, Danielle van Westen, and Anna Falk Delgado. 2018. “Arterial Spin Labeling MR Imaging for Differentiation between High- and Low-Grade Glioma-a Meta-Analysis.” Neuro-Oncology 20 (11): 1450–61.

Komatsu, Katsuya, Masahiko Wanibuchi, Takeshi Mikami, Yukinori Akiyama, Satoshi Iihoshi, Kei Miyata, Toshiya Sugino, et al. 2018. “Arterial Spin Labeling Method as a Supplemental Predictor to Distinguish Between High- and Low-Grade Gliomas.” World Neurosurgery 114 (June): e495–500.

Mutsaerts, Henri J. M. M., Matthias J. P. van Osch, Fernando O. Zelaya, Danny J. J. Wang, Wibeke Nordhøy, Yi Wang, Stephen Wastling, et al. 2015. “Multi-vendor reliability of arterial spin labeling perfusion MRI using a near-identical sequence: Implications for multi-center studies.” NeuroImage 113 (June): 143–52.

Qiao, X. J., B. M. Ellingson, H. J. Kim, D. J. J. Wang, N. Salamon, M. Linetsky, A. R. Sepahdari, et al. 2015. “Arterial Spin-Labeling Perfusion MRI Stratifies Progression-Free Survival and Correlates with Epidermal Growth Factor Receptor Status in Glioblastoma.” AJNR. American Journal of Neuroradiology 36 (4): 672–77.

Sousa, Inês, Pedro Vilela, and Patrícia Figueiredo. 2014. “Reproducibility of the Quantification of Arterial and Tissue Contributions in Multiple Postlabeling Delay Arterial Spin Labeling.” Journal of Magnetic Resonance Imaging: JMRI 40 (6): 1453–62.

Suh, Chong Hyun, Ho Sung Kim, Seung Chai Jung, Choong Gon Choi, and Sang Joon Kim. 2018. “Perfusion MRI as a Diagnostic Biomarker for Differentiating Glioma from Brain Metastasis: A Systematic Review and Meta-Analysis.” European Radiology 28 (9): 3819–31.

Wang, Ning, Shu-Yi Xie, Hui-Ming Liu, Guo-Quan Chen, and Wei-Dong Zhang. 2019. “Arterial Spin Labeling for Glioma Grade Discrimination: Correlations with IDH1 Genotype and 1p/19q Status.” Translational Oncology 12 (5): 749–56.

Yoo, Roh-Eul, Tae Jin Yun, Inpyeong Hwang, Eun Kyoung Hong, Koung Mi Kang, Seung Hong Choi, Chul-Kee Park, Jae-Kyung Won, Ji-Hoon Kim, and Chul-Ho Sohn. 2020. “Arterial Spin Labeling Perfusion-Weighted Imaging Aids in Prediction of Molecular Biomarkers and Survival in Glioblastomas.” European Radiology 30 (2): 1202–11.

Zeng, Q., B. Jiang, F. Shi, C. Ling, F. Dong, and J. Zhang. 2017. “3D Pseudocontinuous Arterial Spin-Labeling MR Imaging in the Preoperative Evaluation of Gliomas.” AJNR. American Journal of Neuroradiology 38 (10): 1876–83.

Zhou, Limin, Yiming Wang, Marco C. Pinho, Edward Pan, Yin Xi, Joseph A. Maldjian, and Ananth J. Madhuranthakam. 2020. “Intrasession Reliability of Arterial Spin-Labeled MRI-Measured Noncontrast Perfusion in Glioblastoma at 3 T.” Tomography (Ann Arbor, Mich.) 6 (2): 139–47.

## References Level of Validation Table - VSI

Batchelor, Tracy T., Elizabeth R. Gerstner, Kyrre E. Emblem, Dan G. Duda, Jayashree Kalpathy-Cramer, Matija Snuderl, Marek Ancukiewicz, et al. 2013. “Improved Tumor Oxygenation and Survival in Glioblastoma Patients Who Show Increased Blood Perfusion after Cediranib and Chemoradiation.” Proceedings of the National Academy of Sciences of the United States of America 110 (47): 19059–64.

Boxerman, J. L., L. M. Hamberg, B. R. Rosen, and R. M. Weisskoff. 1995. “MR Contrast due to Intravascular Magnetic Susceptibility Perturbations.” Magnetic Resonance in Medicine: Official Journal of the Society of Magnetic Resonance in Medicine / Society of Magnetic Resonance in Medicine 34 (4): 555–66.

Chakhoyan, Ararat, Jingwen Yao, Kevin Leu, Whitney B. Pope, Noriko Salamon, William Yong, Albert Lai, et al. 2019. “Validation of Vessel Size Imaging (VSI) in High-Grade Human Gliomas Using Magnetic Resonance Imaging, Image-Guided Biopsies, and Quantitative Immunohistochemistry.” Scientific Reports 9 (1): 2846.

Digernes, Ingrid, Line B. Nilsen, Endre Grøvik, Atle Bjørnerud, Grethe Løvland, Einar Vik-Mo, Torstein R. Meling, et al. 2020. “Noise Dependency in Vascular Parameters from Combined Gradient-Echo and Spin-Echo DSC MRI.” Physics in Medicine and Biology 65 (22): 225020.

Douma, Kim, Marlies Oostendorp, Dick W. Slaaf, Mark J. Post, Walter H. Backes, and Marc A. M. J. van Zandvoort. 2010. “Evaluation of Magnetic Resonance Vessel Size Imaging by Two-Photon Laser Scanning Microscopy.” Magnetic Resonance in Medicine: Official Journal of the Society of Magnetic Resonance in Medicine / Society of Magnetic Resonance in Medicine 63 (4): 930–39.

Emblem, Kyrre E., Christian T. Farrar, Elizabeth R. Gerstner, Tracy T. Batchelor, Ronald J. H. Borra, Bruce R. Rosen, A. Gregory Sorensen, and Rakesh K. Jain. 2014. “Vessel Caliber--a Potential MRI Biomarker of Tumour Response in Clinical Trials.” Nature Reviews. Clinical Oncology 11 (10): 566–84.

Emblem, Kyrre E., Kim Mouridsen, Atle Bjornerud, Christian T. Farrar, Dominique Jennings, Ronald J. H. Borra, Patrick Y. Wen, et al. 2013. “Vessel Architectural Imaging Identifies Cancer Patient Responders to Anti-Angiogenic Therapy.” Nature Medicine 19 (9): 1178–83.

Farrar, Christian T., Walid S. Kamoun, Carsten D. Ley, Young R. Kim, Seon J. Kwon, Guangping Dai, Bruce R. Rosen, Emmanuelle di Tomaso, Rakesh K. Jain, and A. Gregory Sorensen. 2010. “In Vivo Validation of MRI Vessel Caliber Index Measurement Methods with Intravital Optical Microscopy in a U87 Mouse Brain Tumor Model.” Neuro-Oncology 12 (4): 341–50.

Foda, Asmaa, Elias Kellner, Asanka Gunawardana, Xiang Gao, Martin Janz, Anna Kufner, Ahmed A. Khalil, et al. 2022. “Differentiation of Cerebral Neoplasms with Vessel Size Imaging (VSI).” Clinical Neuroradiology 32 (1): 239–48.

Gerstner, Elizabeth R., Kyrre E. Emblem, and Gregory A. Sorensen. 2015. “Vascular Magnetic Resonance Imaging in Brain Tumors During Antiangiogenic Therapy—Are We There Yet?” The Cancer Journal from Scientific American 21 (4): 337.

Gerstner, Elizabeth R., Kyrre E. Emblem, Yi-Fen Yen, Jorg Dietrich, Justin T. Jordan, Ciprian Catana, Kevin Lou Wenchin, et al. 2020. “Vascular Dysfunction Promotes Regional Hypoxia after Bevacizumab Therapy in Recurrent Glioblastoma Patients.” Neuro-Oncology Advances 2 (1): vdaa157.

Guo, Hong, Houyi Kang, Haipeng Tong, Xuesong Du, Heng Liu, Yong Tan, Yizeng Yang, Sumei Wang, and Weiguo Zhang. 2019. “Microvascular Characteristics of Lower-Grade Diffuse Gliomas: Investigating Vessel Size Imaging for Differentiating Grades and Subtypes.” European Radiology 29 (4): 1893–1902.

Heiland, Dieter Henrik, Theo Demerath, Elias Kellner, Valerij G. Kiselev, Dietmar Pfeifer, Oliver Schnell, Ori Staszewski, Horst Urbach, Astrid Weyerbrock, and Irina Mader. 2017. “Molecular Differences between Cerebral Blood Volume and Vessel Size in Glioblastoma Multiforme.” Oncotarget 8 (7): 11083–93.

Kalpathy-Cramer, Jayashree, Vyshak Chandra, Xiao Da, Yangming Ou, Kyrre E. Emblem, Alona Muzikansky, Xuezhu Cai, et al. 2017. “Phase II Study of Tivozanib, an Oral VEGFR Inhibitor, in Patients with Recurrent Glioblastoma.” Journal of Neuro-Oncology 131 (3): 603–10.

Kang, Houyi, Peng Chen, Hong Guo, Letian Zhang, Yong Tan, Hualiang Xiao, Ao Yang, Jingqin Fang, and Weiguo Zhang. 2020. “Vessel Size Imaging Is Associated with IDH Mutation and Patient Survival in Diffuse Lower-Grade Glioma.” Cancer Management and Research 12 (October): 9801–11.

Kang, H-Y, H-L Xiao, J-H Chen, Y. Tan, X. Chen, T. Xie, J-Q Fang, S. Wang, Y. Yang, and W-G Zhang. 2016. “Comparison of the Effect of Vessel Size Imaging and Cerebral Blood Volume Derived from Perfusion MR Imaging on Glioma Grading.” AJNR. American Journal of Neuroradiology 37 (1): 51–57.

Kellner, Elias, Tobias Breyer, Peter Gall, Klaus Müller, Michael Trippel, Ori Staszewski, Florian Stein, et al. 2015. “MR Evaluation of Vessel Size Imaging of Human Gliomas: Validation by Histopathology.” Journal of Magnetic Resonance Imaging: JMRI 42 (4): 1117–25.

Kim, Minjae, Ji Eun Park, Shin Kyo Yoon, Nakyoung Kim, Young-Hoon Kim, Jeong Hoon Kim, and Ho Sung Kim. 2022. “Vessel Size and Perfusion-Derived Vascular Habitat Refines Prediction of Treatment Failure to Bevacizumab in Recurrent Glioblastomas: Validation in a Prospective Cohort.” European Radiology, October. https://doi.org/10.1007/s00330-022-09164-w.

Kim, M., J. E. Park, K. Emblem, A. Bjørnerud, and H. S. Kim. 2021. “Vessel Type Determined by Vessel Architectural Imaging Improves Differentiation between Early Tumor Progression and Pseudoprogression in Glioblastoma.” AJNR. American Journal of Neuroradiology 42 (4): 663–70.

Kiselev, V. G., R. Strecker, S. Ziyeh, O. Speck, and J. Hennig. 2005. “Vessel Size Imaging in Humans.” Magnetic Resonance in Medicine: Official Journal of the Society of Magnetic Resonance in Medicine / Society of Magnetic Resonance in Medicine 53 (3): 553–63.

Kjølby, B. F., L. Østergaard, and V. G. Kiselev. 2006. “Theoretical Model of Intravascular Paramagnetic Tracers Effect on Tissue Relaxation.” Magnetic Resonance in Medicine: Official Journal of the Society of Magnetic Resonance in Medicine / Society of Magnetic Resonance in Medicine 56 (1): 187–97.

Lemasson, Benjamin, Samuel Valable, Régine Farion, Alexandre Krainik, Chantal Rémy, and Emmanuel L. Barbier. 2013. “In Vivo Imaging of Vessel Diameter, Size, and Density: A Comparative Study between MRI and Histology.” Magnetic Resonance in Medicine: Official Journal of the Society of Magnetic Resonance in Medicine / Society of Magnetic Resonance in Medicine 69 (1): 18–26.

Lu-Emerson, Christine, Dan G. Duda, Kyrre E. Emblem, Jennie W. Taylor, Elizabeth R. Gerstner, Jay S. Loeffler, Tracy T. Batchelor, and Rakesh K. Jain. 2015. “Lessons from Anti-Vascular Endothelial Growth Factor and Anti-Vascular Endothelial Growth Factor Receptor Trials in Patients with Glioblastoma.” Journal of Clinical Oncology: Official Journal of the American Society of Clinical Oncology 33 (10): 1197–1213.

Schmiedeskamp, Heiko, Matus Straka, Rexford D. Newbould, Greg Zaharchuk, Jalal B. Andre, Jean-Marc Olivot, Michael E. Moseley, Gregory W. Albers, and Roland Bammer. 2012. “Combined Spin- and Gradient-Echo Perfusion-Weighted Imaging.” Magnetic Resonance in Medicine: Official Journal of the Society of Magnetic Resonance in Medicine / Society of Magnetic Resonance in Medicine 68 (1): 30–40.

Stadlbauer, Andreas, Ilker Eyüpoglu, Michael Buchfelder, Arnd Dörfler, Max Zimmermann, Gertraud Heinz, and Stefan Oberndorfer. 2019. “Vascular Architecture Mapping for Early Detection of Glioblastoma Recurrence.” Neurosurgical Focus 47 (6): E14.

Stadlbauer, Andreas, Max Zimmermann, Gertraud Heinz, Stefan Oberndorfer, Arnd Doerfler, Michael Buchfelder, and Karl Rössler. 2017. “Magnetic Resonance Imaging Biomarkers for Clinical Routine Assessment of Microvascular Architecture in Glioma.” Journal of Cerebral Blood Flow and Metabolism: Official Journal of the International Society of Cerebral Blood Flow and Metabolism 37 (2): 632–43.

Troprès, I., S. Grimault, A. Vaeth, E. Grillon, C. Julien, J. F. Payen, L. Lamalle, and M. Décorps. 2001. “Vessel Size Imaging.” Magnetic Resonance in Medicine: Official Journal of the Society of Magnetic Resonance in Medicine / Society of Magnetic Resonance in Medicine 45 (3): 397–408.

Troprès, I., L. Lamalle, R. Farion, C. Segebarth, and C. Rémy. 2004. “Vessel Size Imaging Using Low Intravascular Contrast Agent Concentrations.” Magma 17 (3-6): 313–16.

Troprès, I., L. Lamalle, M. Péoc’h, R. Farion, Y. Usson, M. Décorps, and C. Rémy. 2004. “In Vivo Assessment of Tumoral Angiogenesis.” Magnetic Resonance in Medicine: Official Journal of the Society of Magnetic Resonance in Medicine / Society of Magnetic Resonance in Medicine 51 (3): 533–41.

Troprès, Irène, Nicolas Pannetier, Sylvie Grand, Benjamin Lemasson, Anaïck Moisan, Michel Péoc’h, Chantal Rémy, and Emmanuel L. Barbier. 2015. “Imaging the Microvessel Caliber and Density: Principles and Applications of Microvascular MRI.” Magnetic Resonance in Medicine: Official Journal of the Society of Magnetic Resonance in Medicine / Society of Magnetic Resonance in Medicine 73 (1): 325–41.

Vejdani Afkham, Behrouz, Sadegh Masjoodi, Mohammad Ali Oghabian, Seyed Roholah Ghodsi, Mohammad Reza Nazem Zadeh, Ebrahim Esmati, Mostafa Farzin, Maziar Gilasi, and Hasan Hashemi. 2019. “Evaluation of Contrast Agent Dose and Diffusion Coefficient Measurement on Vessel Size Index Estimation.” Magma 32 (5): 529–37.

Winfield, J. M., G. S. Payne, and N. M. deSouza. 2015. “Functional MRI and CT Biomarkers in Oncology.” European Journal of Nuclear Medicine and Molecular Imaging 42 (4): 562–78.

Zwick, Stefan, Ralph Strecker, Valerji Kiselev, Peter Gall, Jochen Huppert, Moritz Palmowski, Wiltrud Lederle, et al. 2009. “Assessment of Vascular Remodeling under Antiangiogenic Therapy Using DCE-MRI and Vessel Size Imaging.” Journal of Magnetic Resonance Imaging: JMRI 29 (5): 1125–33.

## References Level of Validation Table - ADC

Bozdağ, Mustafa, Ali Er, Akın Çinkooğlu, and Sümeyye Ekmekçi. 2021. “Diagnostic Role of Apparent Diffusion Coefficient Combined with Intratumoral Susceptibility Signals in Differentiating High-Grade Gliomas from Brain Metastases.” The Neuroradiology Journal 34 (3): 169–79.

Chen, Lihua, Min Liu, Jing Bao, Yunbao Xia, Jiuquan Zhang, Lin Zhang, Xuequan Huang, and Jian Wang. 2013. “The Correlation between Apparent Diffusion Coefficient and Tumor Cellularity in Patients: A Meta-Analysis.” PloS One 8 (11): e79008.

Cindil, Emetullah, Halit Nahit Sendur, Mahi Nur Cerit, Nesrin Erdogan, Filiz Celebi, Nurullah Dag, Emrah Celtikci, Arda Inan, Yusuf Oner, and Turgut Tali. 2022. “Prediction of IDH Mutation Status in High-Grade Gliomas Using DWI and High T1-Weight DSC-MRI.” Academic Radiology 29 Suppl 3 (March): S52–62.

Gihr, Georg, Diana Horvath-Rizea, Elena Hekeler, Oliver Ganslandt, Hans Henkes, Karl-Titus Hoffmann, Cordula Scherlach, and Stefan Schob. 2021. “Diffusion Weighted Imaging in High-Grade Gliomas: A Histogram-Based Analysis of Apparent Diffusion Coefficient Profile.” PloS One 16 (4): e0249878.

Higaki, Toru, Yuko Nakamura, Fuminari Tatsugami, Yoko Kaichi, Motonori Akagi, Yuij Akiyama, Yasutaka Baba, Makoto Iida, and Kazuo Awai. 2018. “Introduction to the Technical Aspects of Computed Diffusion-Weighted Imaging for Radiologists.” Radiographics: A Review Publication of the Radiological Society of North America, Inc 38 (4): 1131–44.

Hu, Ranliang, and Michael J. Hoch. 2021. “Application of Diffusion Weighted Imaging and Diffusion Tensor Imaging in the Pretreatment and Post-Treatment of Brain Tumor.” Radiologic Clinics of North America 59 (3): 335–47.

Hu, Xvlei, Meifeng Xue, Shengyu Sun, Yourui Zou, Jiangping Li, Xiaodong Wang, Xiaoli Liu, and Hui Ma. 2021. “Combined Application of MRS and DWI Can Effectively Predict Cell Proliferation and Assess the Grade of Glioma: A Prospective Study.” Journal of Clinical Neuroscience: Official Journal of the Neurosurgical Society of Australasia 83 (January): 56–63.

Leu, Kevin, Garrett A. Ott, Albert Lai, Phioanh L. Nghiemphu, Whitney B. Pope, William H. Yong, Linda M. Liau, Timothy F. Cloughesy, and Benjamin M. Ellingson. 2017. “Perfusion and Diffusion MRI Signatures in Histologic and Genetic Subtypes of WHO Grade II-III Diffuse Gliomas.” Journal of Neuro-Oncology 134 (1): 177–88.

Liu, Dan, Shuai-Xiang Gao, Hong-Fan Liao, Jing-Mei Xu, and Ming Wen. 2020. “A Comparative Study of 2 Different Segmentation Methods of ADC Histogram for Differentiation Genetic Subtypes in Lower-Grade Diffuse Gliomas.” BioMed Research International 2020 (September): 9549361.

Maynard, John, Sachi Okuchi, Stephen Wastling, Ayisha Al Busaidi, Ofran Almossawi, Wonderboy Mbatha, Sebastian Brandner, et al. 2021. “World Health Organization Grade II/III Glioma Molecular Status: Prediction by MRI Morphologic Features and Apparent Diffusion Coefficient.” Radiology 298 (1): E61.

Minh Thong, Pham, and Nguyen Minh Duc. 2020. “The Role of Apparent Diffusion Coefficient in the Differentiation between Cerebellar Medulloblastoma and Brainstem Glioma.” Neurology International 12 (3): 34–40.

Momeni, Farideh, Razzagh Abedi-Firouzjah, Zahra Farshidfar, Nastaran Taleinezhad, Leila Ansari, Ali Razmkon, Amin Banaei, and Alireza Mehdizadeh. 2021. “Differentiating Between Low- and High-Grade Glioma Tumors Measuring Apparent Diffusion Coefficient Values in Various Regions of the Brain.” Oman Medical Journal 36 (2): e251.

Park, Yae Won, Ji Eun Park, Sung Soo Ahn, Eui Hyun Kim, Seok-Gu Kang, Jong Hee Chang, Se Hoon Kim, Seung Hong Choi, Ho Sung Kim, and Seung-Koo Lee. 2021. “Magnetic Resonance Imaging Parameters for Noninvasive Prediction of Epidermal Growth Factor Receptor Amplification in Isocitrate Dehydrogenase-Wild-Type Lower-Grade Gliomas: A Multicenter Study.” Neurosurgery 89 (2): 257–65.

Soliman, Radwa K., Abdelhakeem A. Essa, Ahmed A. S. Elhakeem, Sara A. Gamal, and Mohamed M. A. Zaitoun. 2021. “Texture Analysis of Apparent Diffusion Coefficient (ADC) Map for Glioma Grading: Analysis of Whole Tumoral and Peri-Tumoral Tissue.” Diagnostic and Interventional Imaging 102 (5): 287–95.

Thust, S. C., S. Heiland, A. Falini, H. R. Jäger, A. D. Waldman, P. C. Sundgren, C. Godi, et al. 2018. “Glioma Imaging in Europe: A Survey of 220 Centres and Recommendations for Best Clinical Practice.” European Radiology 28 (8): 3306–17.

Tsougos, Ioannis, Patricia Svolos, Evanthia Kousi, Konstantinos Fountas, Kyriaki Theodorou, Ioannis Fezoulidis, and Eftychia Kapsalaki. 2012. “Differentiation of Glioblastoma Multiforme from Metastatic Brain Tumor Using Proton Magnetic Resonance Spectroscopy, Diffusion and Perfusion Metrics at 3 T.” Cancer Imaging: The Official Publication of the International Cancer Imaging Society 12 (3): 423–36.

Vermoolen, M. A., T. C. Kwee, and R. A. J. Nievelstein. 2012. “Apparent Diffusion Coefficient Measurements in the Differentiation between Benign and Malignant Lesions: A Systematic Review.” Insights into Imaging 3 (4): 395–409.

Wang, Congxiao, Zhijian Xu, Song Wang, Lijing Peng, Wei Zhang, Xueda Li, Lili Yang, et al. 2021. “Clinical Importance of ADC in the Prediction of 125I in the Treatment for Gliomas.” Journal of Cancer 12 (7): 1945–51.

Zhang, Ying, Yu Lin, Zhen Xing, Shaobo Yao, Dairong Cao, and Wei-Bing Miao. 2022. “Non-Invasive Assessment of Heterogeneity of Gliomas Using Diffusion and Perfusion MRI: Correlation with Spatially Co-Registered PET.” Acta Radiologica 63 (5): 664–71.

## References Level of Validation Table - DTI

Costabile, Jamie D., Elsa Alaswad, Shawn D’Souza, John A. Thompson, and D. Ryan Ormond. 2019. “Current Applications of Diffusion Tensor Imaging and Tractography in Intracranial Tumor Resection.” Frontiers in Oncology 9 (May): 426.

Hempel, Johann-Martin, Sotirios Bisdas, Jens Schittenhelm, Cornelia Brendle, Benjamin Bender, Henk Wassmann, Marco Skardelly, et al. 2017. “In Vivo Molecular Profiling of Human Glioma Using Diffusion Kurtosis Imaging.” Journal of Neuro-Oncology 131 (1): 93–101.

Hu, Ranliang, and Michael J. Hoch. 2021. “Application of Diffusion Weighted Imaging and Diffusion Tensor Imaging in the Pretreatment and Post-Treatment of Brain Tumor.” Radiologic Clinics of North America 59 (3): 335–47.

Jiang, Rui, Fei-Zhou Du, Ci He, Ming Gu, Zhen-Wu Ke, and Jian-Hao Li. 2014. “The Value of Diffusion Tensor Imaging in Differentiating High-Grade Gliomas from Brain Metastases: A Systematic Review and Meta-Analysis.” PloS One 9 (11): e112550.

Leite, Claudia, and Mauricio Castillo. 2016. Diffusion Weighted and Diffusion Tensor Imaging: A Clinical Guide: A Clinical Guide. Thieme.

Liang, Ruofei, Xiang Wang, Mao Li, Yuan Yang, Jiewen Luo, Qing Mao, and Yanhui Liu. 2014. “Potential Role of Fractional Anisotropy Derived from Diffusion Tensor Imaging in Differentiating High-Grade Gliomas from Low-Grade Gliomas: A Meta-Analysis.” International Journal of Clinical and Experimental Medicine 7 (10): 3647–53.

Potgieser, Adriaan R. E., Michiel Wagemakers, Arjen L. J. van Hulzen, Bauke M. de Jong, Eelco W. Hoving, and Rob J. M. Groen. 2014. “The Role of Diffusion Tensor Imaging in Brain Tumor Surgery: A Review of the Literature.” Clinical Neurology and Neurosurgery 124 (September): 51–58.

Seow, Pohchoo, Aditya T. Hernowo, Vairavan Narayanan, Jeannie Hsiu Ding Wong, Nor Faizal Ahmad Bahuri, Chun Yoong Cham, Nor Aniza Abdullah, Khairul Azmi Abdul Kadir, Kartini Rahmat, and Norlisah Ramli. 2021. “Neural Fiber Integrity in High- Versus Low-Grade Glioma Using Probabilistic Fiber Tracking.” Academic Radiology 28 (12): 1721–32.

Sollmann, Nico, Haosu Zhang, Alessia Fratini, Noémie Wildschuetz, Sebastian Ille, Axel Schröder, Claus Zimmer, Bernhard Meyer, and Sandro M. Krieg. 2020. “Risk Assessment by Presurgical Tractography Using Navigated TMS Maps in Patients with Highly Motor- or Language-Eloquent Brain Tumors.” Cancers 12 (5). https://doi.org/10.3390/cancers12051264.

Suh, C. H., H. S. Kim, S. C. Jung, and S. J. Kim. 2018. “Diffusion-Weighted Imaging and Diffusion Tensor Imaging for Differentiating High-Grade Glioma from Solitary Brain Metastasis: A Systematic Review and Meta-Analysis.” AJNR. American Journal of Neuroradiology 39 (7): 1208–14.

Thust, S. C., S. Heiland, A. Falini, H. R. Jäger, A. D. Waldman, P. C. Sundgren, C. Godi, et al. 2018. “Glioma Imaging in Europe: A Survey of 220 Centres and Recommendations for Best Clinical Practice.” European Radiology 28 (8): 3306–17.

Xiong, Ji, Wenli Tan, Jianbo Wen, Jiawei Pan, Yin Wang, Jun Zhang, and Daoying Geng. 2016. “Combination of Diffusion Tensor Imaging and Conventional MRI Correlates with Isocitrate Dehydrogenase 1/2 Mutations but Not 1p/19q Genotyping in Oligodendroglial Tumours.” European Radiology 26 (6): 1705–15.

Zhang, Pengcheng, and Bing Liu. 2020. “Differentiation among Glioblastomas, Primary Cerebral Lymphomas, and Solitary Brain Metastases Using Diffusion-Weighted Imaging and Diffusion Tensor Imaging: A PRISMA-Compliant Meta-Analysis.” ACS Chemical Neuroscience 11 (3): 477–83.

## References Level of Validation Table - MRF

Badve, C., A. Yu, S. Dastmalchian, M. Rogers, D. Ma, Y. Jiang, S. Margevicius, et al. 2017. “MR Fingerprinting of Adult Brain Tumors: Initial Experience.” AJNR. American Journal of Neuroradiology 38 (3): 492–99.

Blank, Peter de, Chaitra Badve, Deborah Rukin Gold, Duncan Stearns, Jeffrey Sunshine, Sara Dastmalchian, Krystal Tomei, et al. 2019. “Magnetic Resonance Fingerprinting to Characterize Childhood and Young Adult Brain Tumors.” Pediatric Neurosurgery 54 (5): 310–18.

Körzdörfer, Gregor, Rainer Kirsch, Kecheng Liu, Josef Pfeuffer, Bernhard Hensel, Yun Jiang, Dan Ma, et al. 2019. “Reproducibility and Repeatability of MR Fingerprinting Relaxometry in the Human Brain.” Radiology 292 (2): 429–37.

Statton, Ben K., Joely Smith, Mary E. Finnegan, Gregor Koerzdoerfer, Rebecca A. Quest, and Matthew Grech-Sollars. 2022. “Temperature Dependence, Accuracy, and Repeatability of T1 and T2 Relaxation Times for the ISMRM/NIST System Phantom Measured Using MR Fingerprinting.” Magnetic Resonance in Medicine: Official Journal of the Society of Magnetic Resonance in Medicine / Society of Magnetic Resonance in Medicine 87 (3): 1446–60.

## References Level of Validation Table - Guidelines

- a Imaging biomarker roadmap (O’Connor et al. 2017)
- b RANO (Wen et al. 2017), iRANO (Ellingson, Wen, and Cloughesy 2017), Standardised Brain Tumor Imaging Protocol (Ellingson et al. 2015)
- c GBM EANO/SNO (Wen et al. 2020), EANO diff. glioma (Weller et al. 2021), EANO glioma (Weller et al. 2017)
- d European survey of adv.MRI (Thust et al. 2018), US survey perfusion imaging (Dickerson and Srinivasan 2016).

Dickerson, E., & Srinivasan, A. (2016). Multicenter Survey of Current Practice Patterns in Perfusion MRI in Neuroradiology: Why, When, and How Is It Performed? AJR. American Journal of Roentgenology, 207(2), 406–410.

Ellingson, B. M., Bendszus, M., Boxerman, J., Barboriak, D., Erickson, B. J., Smits, M., Nelson, S. J., Gerstner, E., Alexander, B., Goldmacher, G., Wick, W., Vogelbaum, M., Weller, M., Galanis, E., Kalpathy-Cramer, J., Shankar, L., Jacobs, P., Pope, W. B., Yang, D., … Jumpstarting Brain Tumor Drug Development Coalition Imaging Standardization Steering Committee. (2015). Consensus recommendations for a standardized Brain Tumor Imaging Protocol in clinical trials. Neuro-Oncology, 17(9), 1188–1198.

Ellingson, B. M., Wen, P. Y., & Cloughesy, T. F. (2017). Modified Criteria for Radiographic Response Assessment in Glioblastoma Clinical Trials. Neurotherapeutics: The Journal of the American Society for Experimental NeuroTherapeutics, 14(2), 307–320.

O’Connor, J. P. B., Aboagye, E. O., Adams, J. E., Aerts, H. J. W. L., Barrington, S. F., Beer, A. J., Boellaard, R., Bohndiek, S. E., Brady, M., Brown, G., Buckley, D. L., Chenevert, T. L., Clarke, L. P., Collette, S., Cook, G. J., deSouza, N. M., Dickson, J. C., Dive, C., Evelhoch, J. L., … Waterton, J. C. (2017). Imaging biomarker roadmap for cancer studies. Nature Reviews. Clinical Oncology, 14(3), 169–186.

Thust, S. C., Heiland, S., Falini, A., Jäger, H. R., Waldman, A. D., Sundgren, P. C., Godi, C., Katsaros, V. K., Ramos, A., Bargallo, N., Vernooij, M. W., Yousry, T., Bendszus, M., & Smits, M. (2018). Glioma imaging in Europe: A survey of 220 centres and recommendations for best clinical practice. European Radiology, 28(8), 3306–3317.

Weller, M., van den Bent, M., Preusser, M., Le Rhun, E., Tonn, J. C., Minniti, G., Bendszus, M., Balana, C., Chinot, O., Dirven, L., French, P., Hegi, M. E., Jakola, A. S., Platten, M., Roth, P., Rudà, R., Short, S., Smits, M., Taphoorn, M. J. B., … Wick, W. (2021). EANO guidelines on the diagnosis and treatment of diffuse gliomas of adulthood. Nature Reviews. Clinical Oncology, 18(3), 170–186.

Weller, M., van den Bent, M., Tonn, J. C., Stupp, R., Preusser, M., Cohen-Jonathan-Moyal, E., Henriksson, R., Le Rhun, E., Balana, C., Chinot, O., Bendszus, M., Reijneveld, J. C., Dhermain, F., French, P., Marosi, C., Watts, C., Oberg, I., Pilkington, G., Baumert, B. G., … European Association for Neuro-Oncology (EANO) Task Force on Gliomas. (2017). European Association for Neuro-Oncology (EANO) guideline on the diagnosis and treatment of adult astrocytic and oligodendroglial gliomas. The Lancet Oncology, 18(6), e315–e329.

Wen, P. Y., Chang, S. M., Van den Bent, M. J., Vogelbaum, M. A., Macdonald, D. R., & Lee, E. Q. (2017). Response Assessment in Neuro-Oncology Clinical Trials. Journal of Clinical Oncology: Official Journal of the American Society of Clinical Oncology, 35(21), 2439–2449.

Wen, P. Y., Weller, M., Lee, E. Q., Alexander, B. M., Barnholtz-Sloan, J. S., Barthel, F. P., Batchelor, T. T., Bindra, R. S., Chang, S. M., Chiocca, E. A., Cloughesy, T. F., DeGroot, J. F., Galanis, E., Gilbert, M. R., Hegi, M. E., Horbinski, C., Huang, R. Y., Lassman, A. B., Le Rhun, E., … van den Bent, M. J. (2020). Glioblastoma in adults: a Society for Neuro-Oncology (SNO) and European Society of Neuro-Oncology (EANO) consensus review on current management and future directions. Neuro-Oncology, 22(8), 1073–1113.
